# Large-scale transcriptomics to dissect 2 years of the life of a fungal phytopathogen interacting with its host plant

**DOI:** 10.1186/s12915-021-00989-3

**Published:** 2021-03-23

**Authors:** Elise J. Gay, Jessica L. Soyer, Nicolas Lapalu, Juliette Linglin, Isabelle Fudal, Corinne Da Silva, Patrick Wincker, Jean-Marc Aury, Corinne Cruaud, Anne Levrel, Jocelyne Lemoine, Regine Delourme, Thierry Rouxel, Marie-Hélène Balesdent

**Affiliations:** 1 Université Paris-Saclay, INRAE, AgroParisTech, UMR BIOGER, 78850 Thiverval-Grignon, France; 2 Génomique Métabolique, Genoscope, Institut François Jacob, CEA, CNRS, Université d’Evry, Université Paris-Saclay, 91057 Evry, France; 3 Genoscope, Institut François Jacob, CEA, Université Paris-Saclay, Evry, France; 4 INRAE, Institut Agro, Univ Rennes, IGEPP, 35653 Le Rheu, France

**Keywords:** *Leptosphaeria maculans*, *Brassica napus*, Fungal biology, Transcriptomics, Pathogenesis, Effectors, Expression waves, Chromatin regulation, Lifestyle transitions

## Abstract

**Background:**

The fungus *Leptosphaeria maculans* has an exceptionally long and complex relationship with its host plant, *Brassica napus*, during which it switches between different lifestyles, including asymptomatic, biotrophic, necrotrophic, and saprotrophic stages. The fungus is also exemplary of “two-speed” genome organisms in the genome of which gene-rich and repeat-rich regions alternate. Except for a few stages of plant infection under controlled conditions, nothing is known about the genes mobilized by the fungus throughout its life cycle, which may last several years in the field.

**Results:**

We performed RNA-seq on samples corresponding to all stages of the interaction of *L. maculans* with its host plant, either alive or dead (stem residues after harvest) in controlled conditions or in field experiments under natural inoculum pressure, over periods of time ranging from a few days to months or years. A total of 102 biological samples corresponding to 37 sets of conditions were analyzed. We show here that about 9% of the genes of this fungus are highly expressed during its interactions with its host plant. These genes are distributed into eight well-defined expression clusters, corresponding to specific infection lifestyles or to tissue-specific genes. All expression clusters are enriched in effector genes, and one cluster is specific to the saprophytic lifestyle on plant residues. One cluster, including genes known to be involved in the first phase of asymptomatic fungal growth in leaves, is re-used at each asymptomatic growth stage, regardless of the type of organ infected. The expression of the genes of this cluster is repeatedly turned on and off during infection. Whatever their expression profile, the genes of these clusters are enriched in heterochromatin regions associated with H3K9me3 or H3K27me3 repressive marks. These findings provide support for the hypothesis that part of the fungal genes involved in niche adaptation is located in heterochromatic regions of the genome, conferring an extreme plasticity of expression.

**Conclusion:**

This work opens up new avenues for plant disease control, by identifying stage-specific effectors that could be used as targets for the identification of novel durable disease resistance genes, or for the in-depth analysis of chromatin remodeling during plant infection, which could be manipulated to interfere with the global expression of effector genes at crucial stages of plant infection.

**Supplementary Information:**

The online version contains supplementary material available at 10.1186/s12915-021-00989-3.

## Background

Fungi play a vital role in global ecosystems. They have highly complex life cycles that often remain poorly understood, due to the multiplicity of fungal species and their behavior. Plant-associated fungi (endophytes, symbionts and phytopathogens) contribute to this complexity and diversity of behavior, as they may have beneficial, neutral or detrimental interactions with their hosts; some are of paramount importance in favoring the growth of plants or their adaptation to the environment, whereas others are highly damaging pathogens of cultivated plants [1].

Many fungal pathogens of annual crops display contrasting infection strategies (e.g., the biotrophs *Blumeria graminis* and *Ustilago maydis*, the hemibiotroph *Colletotrichum higginsianum* or the necrotroph *Botrytis cinerea*), with reproducible short cycles of colonization/sporulation, lasting from hours to weeks, on plant leaves. For such species, high-throughput RNA sequencing has made it possible to describe the stage-specific expression of genes during the few days or weeks over which the infection process occurs, under laboratory conditions. Such studies have identified concerted waves of pathogenicity gene expression during fungal infection [2, 3], sets of genes involved in the switch from asymptomatic to necrotrophic growth in the hemibiotrophic fungus *Zymoseptoria tritici* [4, 5], or four waves of expression for genes encoding putative effectors in *C. higginsianum* [6]*.* Short life cycles, of no more than a few weeks on the host, are compatible with the description of biological and phytopathogenic features in experiments performed in controlled conditions.

In many other models, including symbionts and endophytes, the fungi spend much longer interacting with their host and make use of different pathogenic/symbiotic developmental programs to adapt to different lifestyles during host colonization (this is the case in *Leptosphaeria maculans*, the fungus studied here), different host tissues (e.g., *U. maydis* [7];), or different plant species (e.g., rust species with complex pathogenic cycles including two alternate host species [8];). For these species, RNA-Seq approaches also seem to be the approach of choice for dissecting the entire fungal life cycle and describing the biology of the fungus, together with the sets of genes involved and their regulation. However, the use of RNA-Seq to describe the fungal life cycle is challenging and technically demanding. Even for fungal species that can be grown in axenic media and for which miniaturized pathogenicity assays have been developed, parts of the pathogenicity cycle remain inaccessible and cannot be reproduced in laboratory conditions. It is, therefore, essential to sample and monitor isolates from the wild. The second challenge is developing a sufficiently comprehensive a priori knowledge of all the relevant stages of the fungal life cycle, and a means of sampling all of these stages for analysis. The third challenge is the generation of sufficient amounts of high-quality samples (often corresponding to minute amounts of fungal organisms in large amounts of plant material, or samples obtained blind, without knowing whether the fungus is present in the plant at the time of sampling) for RNA-Seq, to ensure the generation of enough reads for appropriate statistical analyses. The fourth challenge is coping with complex field samples, containing not only the phytopathogen and the host plant, but also many other fungal or bacterial species, necessitating a careful evaluation of the specificity of RNA-Seq mapping before analysis. The final challenge is coping with field samples subject to natural infection in variable environmental conditions, potentially resulting in a high level of heterogeneity in terms of the presence of the fungus and its developmental stage within the tissues, and attaining a biologically relevant continuity when monthly samples are obtained over a whole growing season.

*L. maculans*, a pathogen of rapeseed (*Brassica napus*), has an exceptionally complex life cycle compared to most other fungal phytopathogens attacking annual plants. It can infect different plant tissues, undergo multiple switches from biotrophy to necrotrophy during a lengthy lifespan in plant tissues, and can also live as a saprophyte on plant residues. The epidemiological cycle of *L. maculans* is well described [9, 10]. It begins with the hemibiotrophic colonization of young leaves by spores generated by sexual reproduction (ascospores) in early autumn (October–November) in Europe. The fungus first colonizes the leaf tissues as a biotroph, for a few days or weeks, depending on the climatic conditions, without causing any symptoms. It then induces the development of necrotrophic leaf lesions in which its asexual spores (conidia) are produced. The fungus then migrates, without causing symptoms, from the petiole to the stem, where it lives in the plant tissues, as an endophyte, for several months. Finally, at the end of the growing season (May to July in Europe), it switches back to necrotrophic behavior, inducing the formation of a damaging stem canker that may result in plant lodging. Having completed all these stages of infection on living plant tissues, *L. maculans* then switches to a saprotrophic lifestyle, living on crop residues for up to 3 years. It develops structures for sexual reproduction to create the new inoculum (ascospores) for subsequent seasons on these residues. Due to its length and complexity, only a limited part of the life cycle of *L. maculans* is amenable to laboratory experiments. Most of the RNA-Seq-based time-course experiments published to date were performed during the first 2 weeks of infection, on cotyledons in controlled conditions [11–13], or in one set of stem infection conditions in controlled conditions [14].

In many models analyzing primary leaf infection, RNA-Seq approaches have highlighted the importance of genes encoding small secreted proteins (SSPs), acting as effectors, which often compromise plant defense responses [15]. In *L. maculans*, SSP genes are strongly expressed during cotyledon infection in controlled conditions [11–13], whereas another set of putative effectors and a toxin encoded by a secondary metabolite gene cluster, sirodesmin PL, are recruited during stem infection [14, 16]. To date, 20 effector genes have been characterized in *L. maculans*. These genes include nine “early” effector genes (*AvrLm* genes) [17–25] located in AT-rich regions of the fungal genome enriched in transposable elements (TEs) [26] and eleven “late” putative effector genes, with molecular features similar to those of “early” effectors, expressed during stem colonization [14]. Unlike “early” effectors, these genes are located in gene-rich regions of the genome.

In addition to effector genes, the possible role in virulence of dozens of genes of different functional categories has been studied in *L. maculans*. These genes encode proteins involved in the biosynthesis of two secondary metabolites: abscisic acid (ABA) [27] and the toxin sirodesmin [16], and genes disrupted or silenced in *L. maculans*, 11 of which have been implicated in fungal virulence [28–36]. Again, all these studies were performed in controlled conditions, generally with miniaturized pathogenicity tests on cotyledons, and nothing is known about the involvement of the candidate genes, or of other pathogenicity genes, in other parts of the fungal life cycle.

*L. maculans* has a well-defined bipartite genome consisting of gene-rich, GC-equilibrated regions and large AT-rich regions enriched in transposable elements (TE) but depleted of genes. The AT-rich regions account for 34.8% of the genome and are enriched in H3K9me3 (trimethylation of the lysine 9 residue of histone H3) marks, typical of constitutive heterochromatin in other species, whereas the GC-rich regions are enriched in H3K4me2 (dimethylation of the lysine 4 residue of histone H3) marks, typical of euchromatin [37, 38]. In addition, species-specific genes and genes silenced during axenic growth display an enrichment in H3K9me3 and H3K27me3 (trimethylation of the lysine 27 residue of histone H3 [38]) heterochromatin modifications. The chromatin-based regulation of the expression of “early” effector genes has been experimentally validated for a few *AvrLm* genes in this model [37].

We hypothesized here that, with the use of careful experimental and sampling procedures, it should be possible to use a large-scale transcriptomic approach to decipher the whole of the complex life cycle of *L. maculans* in interaction with its host, in both controlled and field conditions, and over time scales ranging from weeks (controlled condition experiments) to months or years (follow-up of plant infection on leaves and stems for a whole growing season, survival of *L. maculans* on residues remaining on the soil for 1 year). Our objective was, thus, to use RNA-Seq data to describe the complete fungal life cycle and pathogenicity cycle and to identify the genes and functions involved in each stage. We found that about 9% of the genes of the fungus were expressed specifically during host-fungus interactions. A clustering of all the genes involved in the host-fungus interaction led to the detection of eight major waves of gene expression that could be consistently related to the consecutive stages of the *L. maculans* life cycle, to tissue-specific expression, or corresponded to genes recycled for all similar lifestyles at different stages of the fungal life cycle. The regulation of expression was strongly associated with the hosting of genes of the eight waves in heterochromatin landscapes, and heterochromatin dynamics is thought to be instrumental in allowing the recycling of expression for genes from the same wave during all stages of fungal biotrophy.

## Results

### Biological and RNA samples representing all aspects of the life cycle of the fungus in interaction with its host

**Fig. 1:**
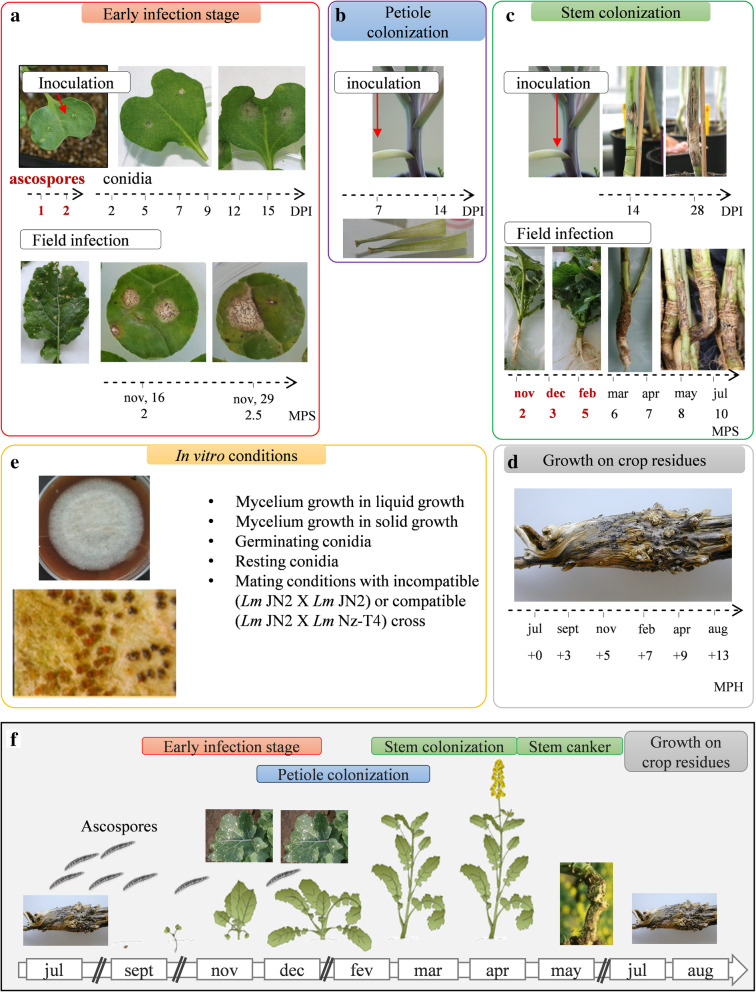
Conditions and samples used for the RNA-Seq study of the *Leptosphaeria maculans* life cycle. Time-course studies are represented by an arrow and the sampling time points are indicated. **a** RNA was extracted from cotyledons infected with ascospores 1 or 2 days post inoculation (DPI). Another time-course study was conducted by inoculation with conidia, with RNA extraction at six time points, from 2 to 15 DPI. Young leaves from naturally infected fields were sampled 2 or 2.5 months post sowing (MPS). **b** Petiole colonization was achieved on petioles inoculated in controlled conditions, with RNA extraction at 7 and 14 DPI. **c** Stem colonization samples were obtained in controlled conditions, at 14 and 28 DPI. Stem base samples from naturally infected fields were collected and used for RNA extraction every 2 to 3 months over an entire growing season. **d** Leftover stem residues were sampled every 2 to 3 months for 1 year after harvest (dates expressed in months post harvest, MPH). **e** We used 10 sets of in vitro growth conditions, based on two different media (liquid or solid) and corresponding to different physiological states (mycelium, sporulation, germinating, and resting conidia, in vitro crossing conditions). The samples highlighted in bold red did not provide enough fungal RNA-Seq reads for statistical analysis. **f** Schematic representation of the colonization stages of oilseed rape by *L. maculans*. The plants are sown in mid-August/early September. During the early infection stage in autumn, the ascospores, generated on crop residues, produce hyphae that colonize the cotyledons/young leaves. Then, the fungus migrates asymptomatically in the petioles and the stems during the winter months. In early summer, *L. maculans* causes stem cankers. Then, *L. maculans* survives on crop residues and produces a new inoculum

The RNA-Seq experiment was designed to generate samples corresponding to all stages of the interaction of *L. maculans* with its host plant, either alive (Fig. 1e–c) or dead (stem residues after harvest; Fig. 1d), in controlled conditions or in field experiments under natural inoculum pressure, over periods of time ranging from a few days to months or years (Fig. 1; For a detailed description of biological samples, see the “Methods” section). As a reference, we included a series of samples grown in axenic conditions promoting a particular fungal developmental stage (mycelium growth, pycnidium differentiation, pseudothecium differentiation, resting conidia, conidial germination) (Fig. 1e), to distinguish between these fundamental biological processes and those specific to interaction with the plant. In total, 102 biological samples, corresponding to 37 different sets of conditions, were subjected to RNA-Seq. Several quality control checks and parameter optimization were required before this heterogeneous dataset could be subjected to statistical analyses. In fine, 32 sets of conditions were retained for statistical analyses, whereas five sets of conditions were excluded because too few fungal reads were available (Additional files 1, 2 and 3: S1 Text, S1 and S2 Tables).

### Quality control analyses highlight the consistency of the RNA-Seq dataset

**Fig. 2:**
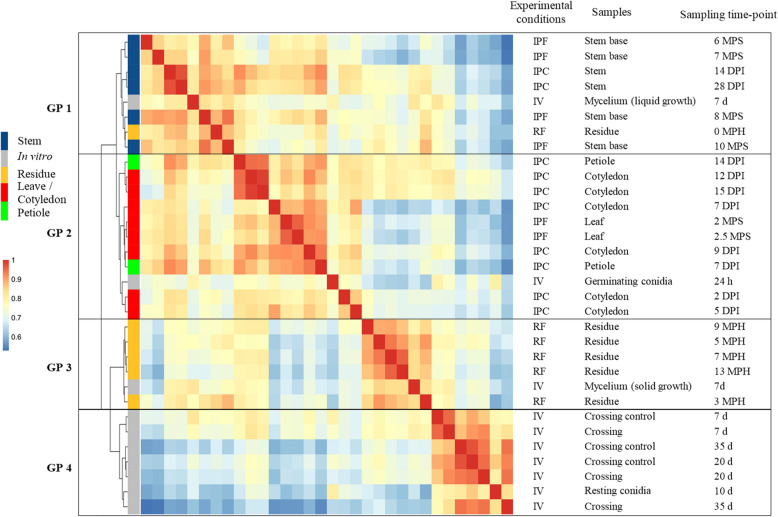
Hierarchical clustering of *Leptosphaeria maculans* gene expression in 32 conditions representing the fungal life cycle. Each gene with an FPKM count > 2 in at least one set of conditions was retained for the correlation analysis. A pairwise Pearson’s correlation analysis was performed between the 32 sets of conditions on Log_2_(FPKM+ 1) values. The resulting Pearson’s correlation matrix was subjected to hierarchical clustering (represented by the tree) with the ward.D2 method. The Pearson’s correlation results are shown on a color scale. Four groups of clustered samples were identified (GP1 to GP4). The experimental conditions are indicated as IPF, in planta field conditions; IPC, in planta controlled conditions; IV, in vitro conditions and RF, residues in field conditions. The infected plant tissues collected or the conditions under which the in vitro samples were obtained are detailed, together with the corresponding time points for sampling (DPI, days post inoculation; MPS, months post sowing; MPH, months post harvest; d, days; h, hours)

Clustering based on pairwise Pearson’s correlation analyses of the 32 sets of conditions identified four groups of clustered conditions (Fig. 2). In each of these four groups, principal component analysis (PCA) showed a low level of variation between biological replicates, except for a few field samples and in vitro crosses (Additional files 4 and 5: S2 Text, S1A-D Fig). The four clusters (Fig. 2) corresponded to the following: (i) the early infection stage on cotyledons and leaves and colonization of the petiole (group 2), (ii) the late infection stage on stems (group 1), (iii) the saprophytic lifestyle (group 3), in controlled conditions or in the field, and (iv) all the in vitro growth conditions promoting the differentiation of pseudothecia and/or pycnidia (group 4).

Within each group, lifestyle provided a second level of sample discrimination, with samples from biotrophic or endophytic stages distinguished from necrotrophic-stage samples (Fig. 2). For example, within group 2, samples displaying necrotrophic behavior on petioles 14 days post infection (DPI) were more closely related to the necrotrophic samples collected from cotyledons 12 and 15 DPI than to the corresponding biotrophic samples (petioles 7 DPI) (Fig. 2). Similarly, in group 1, the residues collected immediately after harvest grouped with the last samples collected from the stem base, even though the residues did not originate from the same growing season, or from the same rapeseed variety, suggesting that the transition from stem necrosis to saprophytic life takes place after harvest (Fig. 2).

Some axenic growth conditions were also grouped with some in planta conditions. Within group 2, germinating conidia grouped with the earliest samples collected from cotyledons, consistent with the use of conidia to inoculate plant tissues in controlled conditions (Fig. 2). Samples grown in axenic culture on solid V8 medium under conditions promoting mycelial growth over sporulation clustered within group 3, indicating that mycelial growth is a major component of the saprophytic lifestyle in the wild. By contrast, mycelium grown in static liquid medium grouped with all conditions in which the fungus colonized stem tissues, possibly reflecting growth under low-oxygen conditions in plant vessels or intercellular spaces.

### Analytical strategy for identifying genes involved in pathogen-plant interactions

**Fig. 3:**
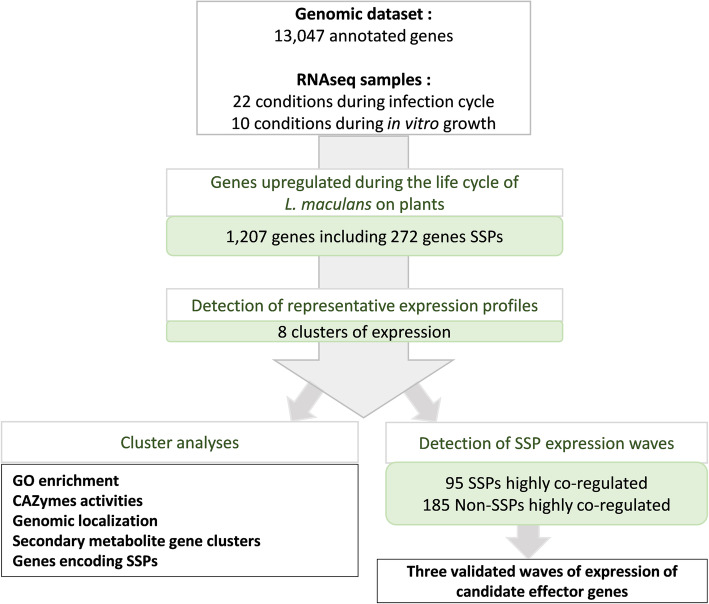
Pipeline used to describe *Leptosphaeria maculans* genes overexpressed in at least one condition in planta. We found that 1207 of the 13,047 annotated genes were overexpressed in at least one of the 22 sets of conditions in planta relative to the 10 sets of in vitro conditions. These genes included 272 predicted to encode small secreted proteins (SSPs). We analyzed the set of 1207 genes to identify the major expression profiles by gene expression clustering. We defined eight expression clusters (clusters 1 to 8). For each cluster, we analyzed expression patterns, functional enrichment, and the genomic localization of the genes. For the specific detection of highly coregulated waves of SSPs, we performed a linear regression analysis of the mean expression value for each SSP subset present in each cluster against the expression of all 13,047 genes. This analysis identified 95 SSP genes and 185 non-SSP genes as highly coregulated genes

For the identification of fungal genes specifically involved in at least one stage of the pathogen-plant interaction, we used the 10 sets of in vitro growth conditions as 10 sets of control conditions, to exclude all genes involved in basic fungal metabolism or life traits other than those associated with plant infection (Fig. 3). We choose very stringent criteria (LogFC > 4; *p* value < 0.01) to focus on genes specifically and robustly overexpressed in planta relative to in vitro control conditions. We analyzed the expression patterns of these genes, their functional annotation, their genomic localization, and their location with respect to histone modifications during axenic growth. We then focused on genes encoding SSPs from the new repertoire generated in this study (Additional file 6: S3 Text). Finally, the expression profiles of co-expressed SSP genes were used as a template for the identification of waves of genes displaying a high level of coregulation (*R*-squared > 0.80) and exemplifying specific pathogenic strategies (Fig. 3).

### Genes overexpressed during interaction with the plant define eight biologically relevant expression clusters

**Fig. 4:**
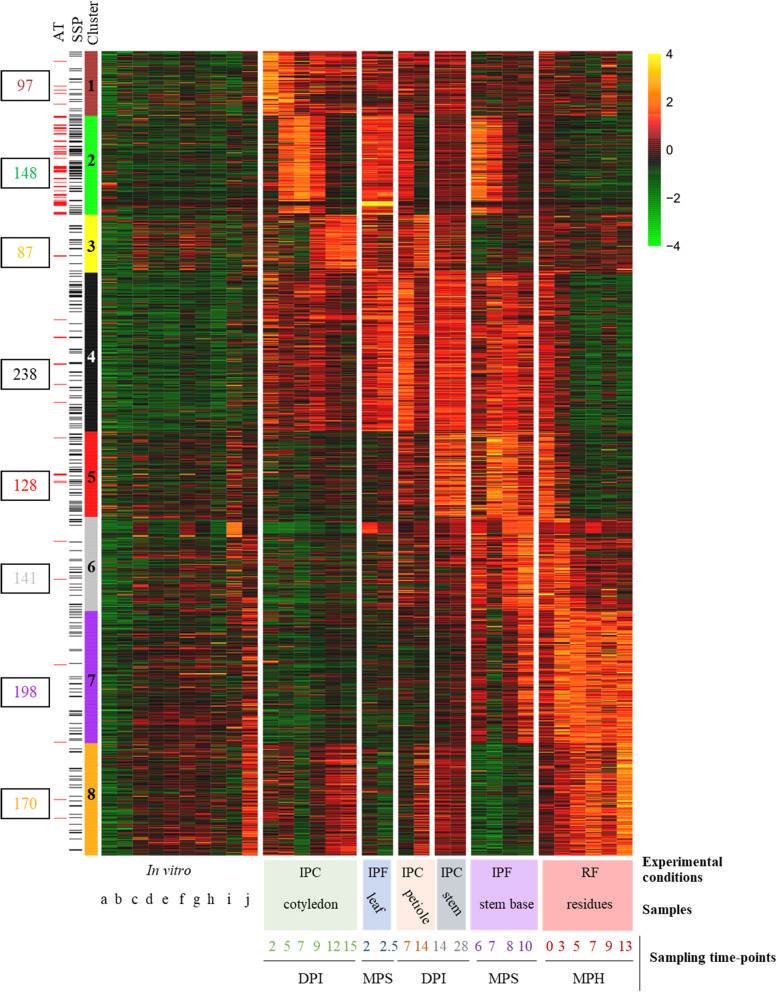
Heatmap obtained by clustering the 1207 *Leptosphaeria maculans* genes upregulated in planta*.* For each set of conditions, we calculated the mean FPKM value of the replicates, which we then subjected to Log_2_ transformation and scaling. The self-organizing map method was used on the Log_2_(FPKM+ 1) values, to define eight expression clusters (clusters 1 to 8) for the 1207 genes. The level of expression ranged from 4 (yellow) to − 4 (green). Genes located in AT-rich regions (AT) or encoding small secreted proteins (SSPs) are indicated by red and black lines, respectively, on the left. The conditions are described in the legend to the *x*-axis of the heatmap. Three sample features are described: (i) the experimental conditions: IPF, in planta field conditions; IPC, in planta controlled conditions; RF, residues in field conditions; (ii) the type of plant tissue sampled; and (iii) the sampling time points (DPI, days post inoculation; MPS, months post sowing; MPH, months post harvest. The ten sets of in vitro conditions are ordered as follows: conidia (a. non-germinating, b. germinating); incompatible mating conditions (c. JN2 x JN2 7 days (d), d. JN2 x JN2 20d, e. JN2 x JN2 35d); compatible mating conditions (f. JN2 x Nz-T4 7d, g. JN2 x Nzt4 20d, h. JN2 x Nz-T4 35d); mycelial growth (i. in liquid medium, j. on solid medium)

Relative to axenic growth conditions, 1207 genes were found to be overexpressed in at least one set of conditions in planta; thus, 9.2% of *L. maculans* genes are specifically mobilized during the interaction of the fungus with its host, alive or dead. Eight expression profiles were identified, referred to hereafter as clusters 1 to 8, defining a complex landscape of expression profiles (Fig. 4). We analyzed the enrichment of each cluster in GO terms, focusing on CAZymes (carbohydrate-active enzymes) associated with degradation activities, to identify the functions and pathways associated with each expression profile (Additional files 7, 8, 9 and 10: S2-S5 Fig).

Clusters 1, 2, and 3 included genes expressed sequentially during cotyledon infection and were representative of three consecutive stages occurring during the 2-week period of cotyledon colonization by *L. maculans*. Cluster 1 encompassed 97 genes highly expressed at the first stages of cotyledon infection, when the conidia germinate and the hyphae penetrate the wounded plant tissues (2–5 DPI). It was enriched in GO terms corresponding to carbohydrate metabolic processes (*p* = 1 × 10^− 2^) and catalytic activities (*p* = 1 × 10^− 3^), including peptidase and hydrolase activities (all *p* ≤ 1 × 10^− 2^). An enrichment in cutinase activity (*p* = 1 × 10^− 2^), specific to this cluster, was also observed (Additional files 7, 8 and 10: S2-S3, S5 Figs).

The 148 genes of cluster 2 displayed peaks of expression during all stages of asymptomatic growth within plant tissues (cotyledon colonization 5–9 DPI, first stage of petiole colonization, first two dates of stem colonization in the field). The genes of this cluster were annotated with few GO terms or functions, but were strongly enriched in SSP genes (*p* = 2 × 10^− 16^; Additional file 8: S3 Fig). Cluster 3 encompassed 87 genes highly expressed during the necrotrophic stage on both cotyledons (9–15 DPI) and petioles, but not during the necrotrophic stem canker stage in the field. This cluster was enriched in carboxylic ester hydrolase activities (*p* = 3 × 10^− 3^) suggesting a role in degradation activities specifically targeting the plant cell wall.

Cluster 4 encompassed 238 genes highly expressed during the shift from biotrophy to necrotrophy in cotyledons (7–9 DPI) and in stem bases infected in the field (7–8 months post sowing, “MPS”). This cluster displayed the strongest enrichment in catalytic processes (*p* = 2 × 10^− 10^) and hydrolase activities (*p* = 1 × 10^− 12^), and a specific enrichment in proteolysis processes (*p* = 2 × 10^− 2^; Additional file 8: S3 Fig). It also displayed the largest number of CAZymes (63 genes; Additional file 10: S5 Fig) targeting different substrates, mostly plant cell-wall components, such as pectin, arabinose, and galactan (Additional file 10: S5 Fig).

Two clusters (cluster 5 and 6) displayed an increase in expression late in stem infection, but differed in terms of the timing of expression and the functions involved. Cluster 5 contained 128 genes highly expressed during the asymptomatic colonization of stems in the field (at 7–8 MPS) and on residues immediately after harvest. It was enriched in catalytic activities (*p* = 1 × 10^− 3^), mostly linked to saccharide degradation, suggesting involvement in activities relating to the uptake of sugar resources (carbon-oxygen lyase activity acting on polysaccharides; hydrolase activity; cellulose binding; Additional file 8: S3 Fig). Cluster 6 contained 141 genes highly expressed during colonization of the stem base in the field, with maximal expression at 10 MPS, corresponding to the time at which stem canker develops. This cluster was enriched in catalytic activities (*p* = 2 × 10^− 4^) but was also linked to cofactor binding (*p* = 3 × 10^− 6^), monooxygenase (*p* = 1 × 10^− 2^), and oxidoreduction activities (*p* = 5 × 10^− 5^), involved in detoxification processes (Additional files 8 and 9: S3 and S4 Figs). The higher proportion of xylanase genes in cluster 6 than in cluster 5 (Additional file 10: S5 Fig) suggests a change in the mode of nutrition during the necrotrophic stage on stems.

Finally, clusters 7 and 8 were specific to saprophytic behavior on stem residues. The 198 genes in cluster 7 displayed an increase in expression from the last sampling on necrotic stems until the last sampling on stem residues 1 year after harvest. This cluster was enriched in genes relating to monooxygenase activities (*p* = 1 × 10^− 4^) and contained the largest number of genes encoding LPMOs (lytic polysaccharide monooxygenases), cellulases, and xylanases. Cluster 8 encompassed 170 genes specifically expressed during all stages occurring on senescent or dead plant tissue, with the highest levels of expression during saprophytic growth on residues. It was also enriched in oxidoreductase processes (*p* = 1 × 10^− 3^), in response to oxidative stresses and oxidant detoxification associated with an enrichment in catalase (*p* = 1 × 10^− 3^) and peroxidase (*p* = 3 × 10^− 3^).

The different clusters of gene expression thus can be designated as follows: “Penetration and establishment” (cluster 1), “Biotrophy” (cluster 2), “Cotyledon and petiole necrotrophy” (cluster 3), “Biotrophy to necrotrophy transition” (cluster 4), “Stem biotrophy” (cluster 5), “Stem necrotrophy” (cluster 6), “Stem canker and saprophytism” (cluster 7), and “Saprophytism” (cluster 8).

### Contribution of candidate pathogenicity genes to fungal life in interaction with the plant

#### Genes involved in fungal pathogenicity in other fungal species

We wondered whether the 9.2% of infection-specific genes were conserved in other phytopathogenic fungi and if we could relate their phase-specific expression pattern with the lifestyle of these fungi. Of the corresponding 1207 proteins, 235 (19.5%) were specific to *L. maculans*. Of the remaining 979 proteins sharing homologies with proteins predicted in other fungal species (Additional file 11: S6 Fig), 316 (32.2%) had matches with hypothetical or predicted proteins, while the remaining ones matched with proteins with an annotated function or domain. The cluster 2 comprised the lowest proportion of proteins with orthologs in other species (64.84%) compared to other clusters, likely due to its high content of species-specific effectors (Additional file 11: S6 Fig). Globally, most hits were with related phytopathogenic Dothideomycete species (genera *Bipolaris*, *Alternaria*, *Pyrenophora*, *Parastagonospora*, etc.), for which only limited RNA-Seq resources are available, preventing us from comparing the clusters of expression of *L. maculans* with theirs. In contrast, a non-negligible number of hits were found with species of the *Colletotrichum* or *Fusarium* genera, and to a lesser extent with *Z. tritici* and *B. cinerea*, species with either an hemibiotrophic or a necrotrophic pathogenicity behavior, for which extensive RNA-Seq data are available. For these species, we identified a very low number of homologous annotated proteins present in the eight expression profiles (Additional file 12: S7 Fig). The cluster 4 contained the highest number of homologous proteins annotated in *Fusarium* and *Colletotrichum* species. They corresponded to catalytic activities such as protease and polysaccharide degradation enzymes. These results suggest that the degrading enzymes involved in the transition from biotrophy to necrotrophy are conserved among the hemibiotrophic fungi.

Several genes previously shown to be involved in fungal pathogenicity were found in one of the eight expression clusters and were mostly associated with biotrophic stages of the interaction. One RALF (rapid alkalinization factor) gene known to favor fungal infection [39] was identified in cluster 1 (Lmb_jn3_05329). Two of the six salicylate hydroxylase genes, known to compromise plant salicylic acid signaling in other models [40], were found in clusters 1 (Lmb_jn3_12582) and 2 (Lmb_jn3_11892). Three of the four LysM-domain containing genes of the genome, known to protect chitin from degradation by plant enzymes or to scavenge chitin oligomers [41], were found in clusters 2 (two genes, Lmb_jn3_00461, Lmb_jn3_10047) and 6 (one gene, Lmb_jn3_04300), and *LysM* genes from cluster 2 matched with *Colletotrichum* spp. *LysM* genes expressed in planta. Three isochorismatase genes (Lmb_jn3_11641, Lmb_jn3_10045, Lmb_jn3_06552), interfering with plant salicylate and jasmonate defense signaling in other phytopathogens, were present in clusters 1, 4, and 6, respectively. Last, all the proteins involved in the biosynthesis of ABA in *B. cinerea* matched *L. maculans* proteins annotated in the ABA biosynthesis cluster, but the ABA genes were not expressed in planta in *B. cinerea* while they are expressed in the biotrophy cluster 2 in *L. maculans* (Additional file 12: S7 Fig). Finally, one of the two genes encoding NEP (necrosis and ethylene-inducing protein), frequently associated with necrotrophic behavior [42], was found to be overexpressed in planta, and belonged to cluster 4 (Lmb_jn3_10035).

#### Secondary metabolite gene clusters

The genome of *L. maculans* contains 22 genes predicted by SMURF tools to encode secondary metabolite enzymes (SM) and annotated as “polyketide synthase” (PKS, 11) “non-ribosomal peptide synthetase” (NRPS, 5), or “others” (dimethylallyltryptophan synthase, 1; NRPS-like, 4; PKS-like, 1). Seven of the 1207 genes in the eight expression clusters encoded SM enzymes from the PKS or NRPS family. Four of these genes were highly expressed during necrotrophic stages of the life cycle within the stem (cluster 6), whereas the other three were associated with clusters 2, 4, and 5 (Additional file 13: S3 Table).

Among the SM genes analyzed in previous studies [27, 43, 44], the PKS gene involved in abscisic acid (ABA) synthesis was linked to biotrophic behavior (cluster 2), whereas genes involved in the synthesis of the toxins sirodesmin PL and phomenoic acid were associated with stem necrotrophy, together with one other PKS and one NRPS-like gene (cluster 6) (Additional file 13: S3 Table). Secondary metabolite enzymes encode an SM backbone that may be modified by tailoring enzymes, forming complex secondary metabolite gene clusters (SMGC). Here, only three of the seven SM enzyme genes overexpressed in planta showed coregulation with all or part of surrounding genes of their SMGC: (i) the PKS responsible for ABA production (all genes of the ABA SMGC being included in cluster 2), (ii) the NRPS responsible for sirodesmin PL production (16 of the 25 genes of the SMGC included in cluster 6), and (iii) an unknown NRPS-like gene, co-expressed with nine other surrounding genes also included in cluster 6 (Additional file 14: S8 Fig).

#### Genes involved in *L. maculans* pathogenicity

Thirty-six of the 52 genes previously identified as candidate pathogenicity genes in *L. maculans* (Additional file 15: S4 Table) did not belong to the SM or avirulence effector gene (see below) categories. Fifteen of these genes were identified within a single cluster (Additional file 15: S4 Table): cluster 1 (2 genes), cluster 2 (5 genes), cluster 3 (1 gene), cluster 4 (4 genes), cluster 5 (2 genes), or cluster 7 (1 gene). Nine had previously been selected as putative pathogenicity genes on the basis of their overexpression during infection, for inactivation with CRISP-cas9, but the inactivation of these genes individually resulted in no visible pathogenicity defect at the cotyledon stage [45] (Additional file 15: S4 Table). Only one gene, encoding a 3-ketoacyl-thiolase (cluster 1), was shown to be involved in pathogenicity, but the mutant also displayed impaired in vitro growth and germination [28] (Additional file 15: S4 Table). The other five genes have not been validated by functional approaches and are, thus, still considered to be candidate pathogenicity-related genes. Four of these genes (three GH genes and one ABC transporter) were included in the late expression clusters 4, 5, and 7, suggesting that they may be bona fide pathogenicity genes (Additional file 15: S4 Table).

By contrast, another 21 putative pathogenicity genes did not belong to any of the expression clusters. Nineteen had previously been analyzed in functional studies (knockout or silenced mutants), and 10 were shown to induce a loss or reduction of pathogenicity following inactivation (Additional file 15: S4 Table). However, the inactivation of six of these genes also induced defects in growth, sporulation, or germination [30, 33, 36, 45, 46], suggesting a general effect on different stages of the fungal life cycle with no specific overexpression during interactions with the plant.

#### Validated or candidate SSP effectors of *L. maculans*

SSPs have been identified in *L. maculans* and were classified as “early” effectors (including all known avirulence proteins, inducing an avirulence phenotype when matching the corresponding resistance genes) or “late” candidate effectors by Gervais et al. [14]. The role of avirulence proteins as effectors suppressing plant defense responses or increasing/decreasing the size of cotyledon lesions has been investigated in a few cases following comparisons of near-isogenic *L. maculans* isolates, gene silencing or complementation (Additional file 15: S4 Table, [21, 22, 47, 48]). The most recently published version of the genome assembly of *L. maculans* isolate JN3 ([49]; Additional file 16: S4 Text) included a number of major changes. We therefore produced a new repertoire of genes encoding putative SSPs, encompassing 1070 genes (8.2% of the entire *L. maculans* gene set) (Additional files 6, 17, 18 and 19: S3 Text, S9 Fig, S5 and S6 Tables).

**Fig. 5:**
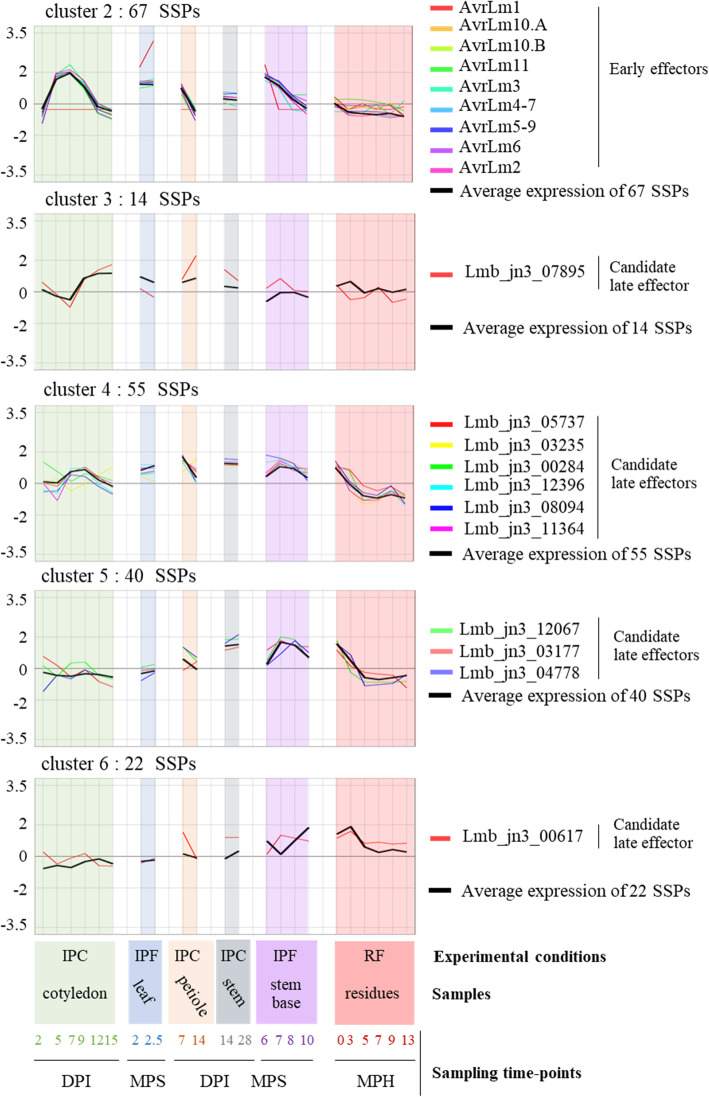
Expression of known and putative effectors of *Leptosphaeria maculans* within the eight expression clusters. The scaled Log_2_(FPKM + 1) expression values for the nine avirulence effector (*AvrLm)* genes, and the eleven candidate late effector genes shown to be upregulated in at least one set of conditions in planta are represented and grouped according to cluster assignment. The mean scaled Log_2_(FPKM + 1) values of all the small secreted protein (SSP) genes in each cluster are indicated (black bold curve). The total number of SSP genes in each cluster is indicated. The conditions are described in the legend to the *x*-axis. Three sample features are described: (i) the experimental conditions: IPF, in planta field conditions; IPC, in planta controlled conditions; RF, residues in field conditions; (ii) the type of plant tissue sampled; and (iii) the sampling time points (DPI, days post inoculation; MPS, months post sowing; MPH, months post harvest)

All expression clusters displayed a significant enrichment in genes encoding SSPs (Additional files 8: S3 Fig). We found that 272 of the 1207 genes upregulated during the *L. maculans* interaction with *B. napus* (22.5%) encoded SSPs. This enrichment was limited in clusters 1, 3, 6, 7, and 8, which contained only 14–17% of SSP-encoding genes (0.013 < *p* < 3.10^− 5^), but three other clusters displayed a much higher level of enrichment in genes encoding SSPs: cluster 4 (23.1%, *p* = 7 × 10^− 16^), cluster 5 (31.2%, *p* = 2 × 10^− 16^), and cluster 2 (45.3%, *p* = 2 × 10^− 16^). Interestingly, both clusters 2 and 5 are associated with biotrophic behavior of the fungus. All the currently known *AvrLm* genes belonged to the “Biotrophy” cluster (cluster 2) (Additional file 15: S4 Table). This cluster displayed sequential rounds of overexpression/repression, with three peaks of overexpression occurring during the three biotrophic stages (5–9 DPI on cotyledons, leaf infection in the field; 7 DPI on petioles; 6–7 MPS in field plant stems) (Fig. 5; Additional file 20: S10 Fig).

The ten “late” effectors, expressed during stem colonization in controlled conditions [14], were scattered over four different clusters (3, 4, 5, 6), along with a series of other genes encoding SSPs (cluster 3, 14 SSPs; cluster 4, 55 SSPs; cluster 5, 40 SSPs and cluster 6, 22 SSPs) (Fig. 5). The “late” effectors in clusters 5 and 6 were specific to stem colonization (and the first stages of saprotrophy on residues), but those in clusters 3 and 4 were also expressed during cotyledon infection. These “late” effector candidates were associated with either biotrophic behavior on stems (cluster 5) or necrotrophic behavior on cotyledons and stems (clusters 3 and 6).

Clusters 1, 7, and 8 were also found to be enriched in genes encoding SSPs (*p* value < 5 × 10^− 3^). They contained 16, 33, and 24 SSP genes, respectively. Candidate effectors may, therefore, be mobilized very early in colonization (cluster 1) and during saprophytic life (clusters 7 and 8) (Additional file 20: S10 Fig).

Additional RNA-Seq samples (ascospores germinating on unwounded cotyledons 24 and 48 h post inoculation; stem base samples 2, 3 and 5 MPS) were available. They provided too few *L. maculans* reads for statistical analyses, but these samples allowed us to confirm that genes belonging to cluster 2, and “early” effector genes in particular, were among the genes most strongly expressed both during ascospore germination on cotyledons and very early stages of stem colonization in winter (Additional files 21 and 22: S5 Text, S7 Table).

### Histone methylation associated with gene regulation

**Fig. 6:**
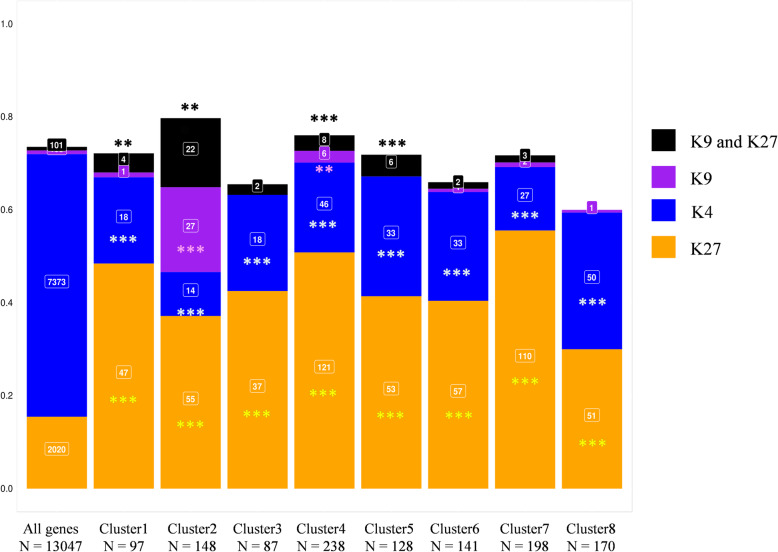
Association of *Leptosphaeria maculans* genes upregulated in planta with the histone modification marks. The genome-wide histone map was generated by ChIP-Seq analysis during axenic growth [38]. The proportion of genes associated with any of the histone modifications H3K4me2, H3K9me3, and H3K27me3 throughout the entire genome was used as a reference set for detecting the over- or under-representation of specific methylation marks in the eight gene clusters overexpressed in planta, in chi-squared tests (***: *p* < 0.001, **: *p* < 0.01). K4, H3K4me2; K9, H3K9me3; K27, H3K27me3

All cloned avirulence genes of *L. maculans* identified to date are associated with H3K9me3. Furthermore, H3K9me3 and H3K27me3 domains are significantly enriched in genes encoding effectors, regardless of their expression [38]. During axenic culture, 7373 *L. maculans* genes are associated with H3K4me2, 104 with H3K9me3, 2020 with H3K27me3 and 101 with the two heterochromatin histone modifications [38]. We combined the genome-wide histone maps previously generated in vitro with our analysis of gene expression during infection, to determine whether (i) all genes, (ii) only (putative) effector genes, or (iii) only certain subsets of genes upregulated in planta were associated with a particular chromatin landscape. H3K4me2-domains encompass 56% of *L. maculans* genes, but only 20% of the genes upregulated in planta were associated with H3K4me2 (*χ*^2^ test = 662; *p* < 2 × 10^− 16^). By contrast, the genes upregulated in planta were significantly enriched in genes located in an H3K27me3 domain, as 44% of the upregulated genes were located in such a domain (*χ*^2^ test = 749, *p* < 2 × 10^− 16^). Strikingly, only 205 genes were located in H3K9me3 or H3K27me3 domains in vitro, but 85 of these genes were upregulated in planta. Thus, regardless of the wave of expression concerned, the genes upregulated in planta were enriched in genes associated with heterochromatin in vitro. Only cluster 2 was significantly enriched in genes associated with the H3K9me3 histone modification (Fig. 6). Interestingly, all clusters of genes upregulated in planta relative to axenic culture were enriched in H3K27me3-associated genes in vitro*,* whereas these clusters contained significantly fewer genes located in H3K4me2-domains in vitro than the rest of the genome (Fig. 6). The integration of RNA-Seq and ChIP-Seq data revealed that the genes upregulated in planta were not randomly located in the genome of *L. maculans*, with H3K4me2-domains significantly depleted of genes upregulated during rapeseed infection, and both types of heterochromatin domains significantly enriched in such genes.

### The co-expression of effector genes defines waves of coregulated gene expression

Almost all the expression clusters were enriched in genes encoding SSPs. However within each cluster, the genes encoding SSPs could have different expression profiles, due to the clustering method (Additional file 20: S10 Fig). We aimed here to increase the robustness of the detection of SSP expression waves. We defined eight SSP reference expression profiles by calculating the mean levels of expression for SSP genes within the eight clusters. We selected genes displaying significant coregulation (SSP and non-SSP genes), by analyzing the linear regression between the expression patterns of the entire set of 13,047 genes and that of each SSP reference expression profile. Genes significantly co-expressed with the reference expression wave tested (*R*^2^ > 0.80, *p* < 0.05) were included in the new SSP expression waves and were considered to be “highly coregulated genes.”

**Fig. 7:**
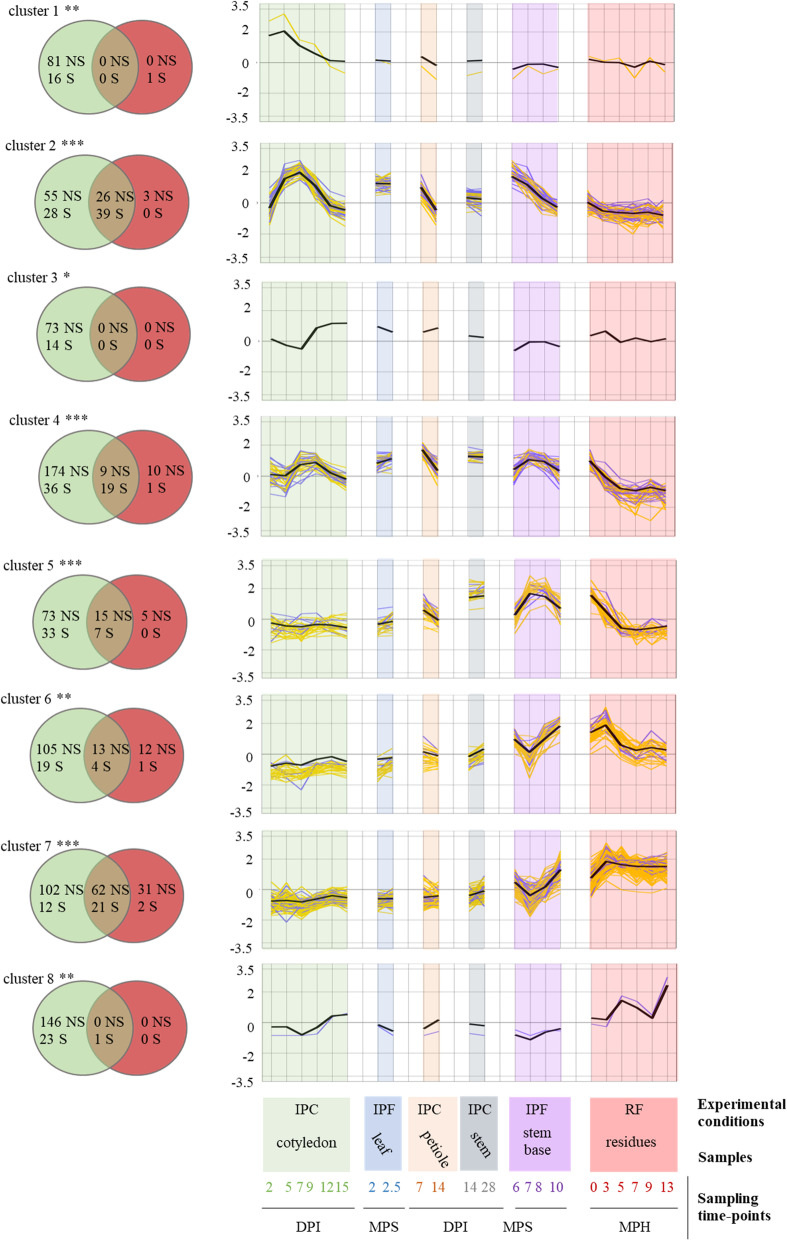
Identification of highly coregulated *Leptosphaeria maculans* genes based on mean SSP gene expression in planta*.* The mean scaled Log_2_(FPKM + 1) values for small secreted protein (SSP) genes in each cluster (clusters 1 to 8) were used to create the eight reference expression profiles (black bold curve). The 22 sets of biological conditions used to average SSP gene expression are described in the legend to the *x*-axis. Three sample features are described: (i) the experimental conditions: IPF, in planta field conditions; IPC, in planta controlled conditions; RF, residues in field conditions; (ii) the type of plant tissue sampled; and (iii) the sampling time points (DPI, days post inoculation; MPS, months post sowing; MPH, months post harvest. A linear regression analysis was performed to determine the correlation between the scaled Log_2_(FPKM+ 1) value for the whole set of genes in the 22 sets of conditions and these eight reference expression profiles. A gene was considered to be highly correlated if its expression fitted the reference expression profile (in black) with an *R*-squared value > 0.80 and a corrected *p* value < 0.05. The expression values of the resulting highly correlated gene subsets (SSP in blue and non-SSP in yellow) are plotted. The Venn diagrams show the number of genes (S: SSP, NS: Non-SSP) present in each cluster (green circles) and the number of genes identified as highly correlated with the corresponding expression profile (red circles). The asterisks indicate the significance of the SSP enrichment in the initial cluster (***: *p* < 0.001, **: *p* < 0.01)

For clusters 1, 3, and 8, no highly coregulated genes could be identified (Fig. 7). For the other five clusters, waves of highly coregulated genes were identified, each containing five to 39 highly coregulated SSP genes (including 90 SSP genes from the 178 previously assigned to these clusters), together with 186 highly coregulated genes encoding other types of proteins (Fig. 7). Two waves of expression, corresponding to clusters 5 and 6, displayed coregulation of a limited number of SSP genes (seven and five, respectively, i.e., 17% of the SSP genes of these clusters), together with 20 and 25 genes encoding other proteins, respectively. However, only 15 (cluster 5) and 13 (cluster 6) of these genes belonged to the clusters (Fig. 7), suggesting that additional coregulated genes are either expressed in axenic conditions, or do not display specific regulation during plant infection. For clusters 2, 4, and 7, a higher proportion of highly coregulated SSP genes was found, with 58% (exact confidence interval [45–70%]), 34% ([22–48%]), and 63% ([45–79%]) of the SSP genes of the clusters retained in the waves of highly coregulated genes, respectively (Fig. 7). Thus, clusters 2 and 7 displayed the highest proportion of highly coregulated SSP genes, suggesting that the regulation of expression is much tighter in these two clusters than in the others. The highly coregulated genes in the “Biotrophy” wave (contained in cluster 2) included 39 SSPs, 23 (59%) of which displayed no sequence matches to the NCBI nr protein database (Additional file 23: S8 Table) and could be considered *L. maculans*-specific effectors. These genes also included other genes mentioned above, such as the genes of the ABA cluster, providing additional support for their role in the biotrophic parts of the interaction. The highly coregulated genes in the “Stem canker and necrotrophy” wave included most of the SSP genes of the cluster, but only two of these genes (9.5%) were specific to *L. maculans*. This wave also included a large number of genes (24) encoding CAZymes.

## Discussion

In this paper, we used large-scale transcriptomic analyses to decipher the complex life cycle of a phytopathogenic fungus, to identify genes paramount for its interactions with the host plant and to shed light on the underlying regulatory mechanisms. In particular, given that many parts of the *L. maculans* life cycle remain poorly described, we focused on how the fungus copes with its different lifestyles, their timing and the transitions between them, and the regulation of gene expression throughout the long pathogenic/saprophytic life of the fungus. We generated RNA-Seq data for biological samples corresponding to the main relevant stages of infection in controlled conditions or in field conditions, and at different time scales (from days to years). Using this large dataset, we then focused on sets of genes mobilized exclusively when the fungus interacts with the plant. We used 10 different sets of axenic growth conditions promoting different aspects of the fungal life cycle (e.g., vegetative growth, asexual sporulation, sexual reproduction) as basic expression features for the identification of pathogenicity genes per se, contrasting with the common practice of using only a few axenic conditions as controls [7, 13, 14] or performing pairwise comparisons of the infection time course [11]. We dealt with the variable number of fungal reads between samples and the diverse in vitro conditions, by applying very stringent conditions to determine whether a gene was specifically overexpressed (LogFC> 4, *p* < 0.01). With these criteria, we showed that 1207 genes (less than 10% of the genes of the fungal genome) were overexpressed during interactions with the living or dead plant relative to axenic conditions. These genes may be considered the maximal set of genes mobilized by the fungus to infect the plant without involvement in basal biological processes. These 1207 upregulated genes were consistently grouped into eight expression profiles, some of which displayed strong coregulation of a number of the genes within the cluster. The sequential expression profiles are consistent with the associated pathogenicity functions, confirming the relevance of this RNA-Seq study performed over a large timescale.

### Transcriptome-based description of a complex fungal life cycle

**Fig. 8:**
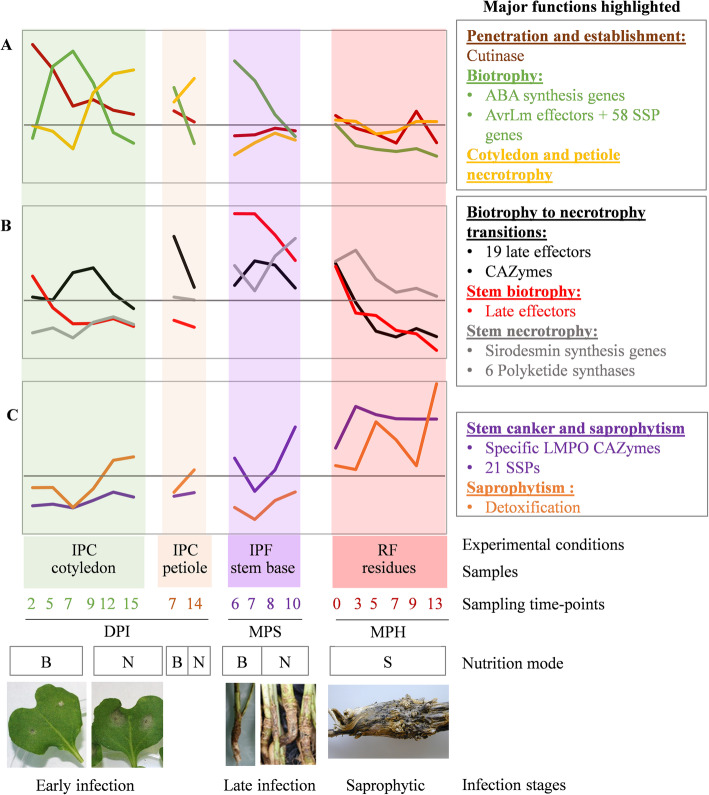
Overview of the eight expression profiles detected during the infection cycle of *Leptosphaeria maculans.* Each curve represents the mean expression value for the genes making up the eight clusters described in this study. The eight profiles are divided into three different plots, depending on the timing of their major expression peaks: (**a**) early infection and colonization of cotyledons, (**b**) late colonization of stems, (**c**) development on crop residues. The characteristics associated with each sample are indicated: mode of nutrition (B: biotrophy, N: necrotrophy, S: saprotrophy); the experimental conditions: (i) IPF, in planta field conditions; IPC, in planta controlled conditions; RF, residues in field conditions; (ii) the type of plant tissue sampled; and (iii) the sampling time points (DPI, days post inoculation; MPS, months post sowing; MPH, months post harvest). We did not include field-infected leaves and the stem infected in controlled conditions, to prevent redundancy and generate a simplified model. The major gene functions identified in each gene cluster are shown on the right

Our RNA-Seq data highlighted and strengthened our knowledge of the biology of the fungus all along its life cycle, including for “obscure parts” of this cycle, as follows (Fig. 8):

(i) Ascospores, which are difficult to generate for pathogenicity tests in controlled conditions, are known to be the main source of infection in the field, germinating within a few hours to produce a hypha that penetrates leaf tissues via stomata or wounds [50]. We found that germinating hyphae very rapidly (2 DPI) produced “early” effector transcripts (including known AvrLm effectors), detected only 5–7 DPI as a massive peak of expression when inoculations were performed with conidia ([11, 13, 14], this study). This result is consistent with microscopy-assisted RNA-Seq analyses in other models, which have suggested that a few hyphal tips produce early effectors to counter the initial defense reactions of the plant upon contact [4, 51]. This suggests that, like other plant-associated fungi, *L. maculans* manipulates the plant as soon as it enters the tissues, to initiate its own growth within plant tissues, without causing symptoms.
(ii) Following the infection of leaves or cotyledons and primary leaf spot development, the hyphae grow within the intercellular spaces within tissues in advance of the leaf spots towards and within the petiole and then enter the stem tissues [52–54]. Our RNA-Seq data highlight the kinetics of this process and the lifestyle transitions occurring during cotyledon infection, with three successive waves of gene expression related to different stages of infection (penetration, biotrophy, and necrotrophy). Consistent with the biological data, the pattern of gene expression during petiole colonization was similar to that in cotyledons, with a symptomless stage during which the same set of genes was mobilized in cotyledons or petioles (Fig. 8).
(iii) We also found that isolates infecting leaves in the field had a pattern of gene expression similar to that of a reference isolate used to inoculate cotyledons in controlled conditions at 7 and 9 DPI, demonstrating that infections reproduced with routine protocols in controlled conditions can accurately mimic some stages of field infection.
(iv) The monitoring of stem samples for a full year in field conditions provided new information about the dynamics of stem colonization and necrosis in the field. We found that the fungus was present very early in the stem base tissues, with fungal reads detectable as early as December, as little as 1 to 2 months after the initial leaf infection. However, levels of fungal transcriptomic activity remained very low between December and February, and it was only at the end of winter (from March on) that the fungus displayed a quantifiable, steady increase in transcriptomic activity.
(v) Fungal life within stem tissues, over a period of 8 months, was characterized by complex patterns of gene expression, with some of the genes concerned expressed exclusively during stem colonization, highlighting the lengthy transition from biotrophy to necrotrophy leading to stem canker (Fig. 8). The gene expression patterns observed for stem infection at 6 and 7 months post sowing (March–April) were very similar to those observed for biotrophic stem infection in controlled conditions at 14 and 28 DPI, again demonstrating that controlled conditions adequately reproduce at least one stage of stem colonization in the field.
(vi) The fungus has been reported to survive on residues for between 2 and 4 years, depending on the climatic conditions [50]. However, we currently know nothing about how *L. maculans* survives as a saprobe on residues. A recent metabarcoding study showed that *L. maculans* was the predominant fungal species on rapeseed residues, on which many other fungal species, including other phytopathogens were also present, highlighting complex interplay between the species [55]. Our RNA-Seq analysis of residues left on the soil for 1 year confirmed these findings and systematically revealed transcriptomic activity of *L. maculans* on the residues, with a mean of 22% of the total RNA-Seq reads corresponding to *L. maculans* throughout the year, but with seasonal variations.

### Expression profiles and characteristics of genes specifically involved in interaction with the plant

We observed successive or partly overlapping expression profiles, highlighting various life traits or feeding strategies specific to a particular lifestyle or colonized tissue (cluster 1, cluster 2, cluster 4, cluster 8), or with a combination of lifestyle/tissue specificities (cluster 3, cluster 5, cluster 6, cluster 7) (Fig. 8). For example, necrotrophic behavior is tissue-specific, with genes expressed during cotyledon or petiole necrosis (cluster 3) or in stem tissues at the end of the growing season (cluster 6; Fig. 8). By contrast, cluster 2 is involved in biotrophic behavior, whatever the tissue colonized.

The set of 1207 genes specifically expressed during interaction with the plant is strongly enriched in candidate effectors, with enrichment in effectors observed for all expression clusters. As shown for cotyledon infection in other studies [13], and expanded here to other biotrophic or necrotrophic stages of plant infection, biotrophic behavior was associated with enrichment in very few, if any GO terms, whereas biotrophy-necrotrophy transitions were strongly enriched in genes involved in catalytic processes and CAZymes. Strict necrotrophic behavior within stems is associated with an overrepresentation of PKS-encoding genes (including the sirodesmin biosynthesis gene cluster) relative to other necrotrophic clusters.

The fungus expresses a set of 148 genes in all biotrophic/endophytic/asymptomatic stages of colonization (cluster 2). Being based on the annotated reference genome, this amount is probably under-estimated when considering field isolates that may have a different repertoire of effector genes. This would be mostly the case for genes of cluster 2 embedded in TE-rich regions and known to be prone to presence/absence polymorphism in field populations. Interestingly, we found that the expression of these genes was up- and down-regulated on multiple occasions during the interaction with the plant. The recycling of the same set of genes for a similar lifestyle highlights the key role played by this set of genes in establishing symptomless growth within the plant, with at least one set of stem-specific genes (cluster 5) also associated with biotrophic behavior, but only during stem colonization. Cluster 2 (and, to a lesser extent, cluster 5) was found to be strongly enriched in genes encoding candidate effectors, which are generally thought to act by compromising plant immunity [15]. One of these candidate effectors, AvrLm10A, has been shown to compromise leaf symptom development [23], and two others, *AvrLm4–7* and *AvrLm1*, interfere with SA and ET signaling [47, 48]. In addition, cluster 2 includes genes reported in various models to be involved in the suppression of plant defense responses (salicylate hydroxylase or the ABA cluster [27, 56, 57];) or the scavenging of chitin oligomers, such as the LysM genes [41]. This strongly suggests that cluster 2 plays an important role in hiding the fungus from the plant surveillance machinery, or in compromising or suppressing plant defense responses to various extents.

Our findings suggest that the previous classification of effectors as “early” or “late” [14] is not relevant. “Early” effector genes were found to be expressed on several occasions during symptomless cotyledon infection and colonization, the symptomless colonization of petioles, and during at least 3 months of symptomless growth in the tissues of the stem base. Similarly, the “late” effector genes identified by Gervais et al. [14] and thought to be specific to stem colonization according to experiments in controlled condition, were actually found in four different expression clusters, only one of which was specific to biotrophic behavior in stem tissues (cluster 5, Fig. 8).

Importantly, cluster 2 contains all the genes encoding avirulence proteins identified to date in *L. maculans*. During leaf or cotyledon infection, these avirulence proteins can be recognized by the products of the major resistance genes (*Rlm* genes) of the plant, resulting in complete resistance to avirulent isolates. The re-use of these same genes at other stages of plant colonization strongly suggests that recognition can also take place during the systemic growth of the fungus within petioles or stems, resulting in efficient resistance at these stages of colonization, provided that the *Rlm* genes are constitutively expressed in all plant tissues. In addition to the nine already cloned avirulence genes, cluster 2 contains 58 additional SSP genes (39 of which are highly coregulated with all *AvrLm* genes), which are candidates of choice for the screening of genetic resources for novel resistance genes.

We also found that effector gene expression was not restricted to interaction with the living plant. Genomic studies on fungal saprotrophs focus on CAZymes and class-II peroxidases [58]. We show here that *L. maculans* recruits a typical cocktail of CAZymes, including the lytic polysaccharide monooxygenases (LPMO) involved in wood decay, but also peroxidases, cytochrome P450, and stress response genes, during 1 year of life on crop residues. In addition, cluster 7 encompasses 21 SSP genes specifically expressed during saprophytic growth on stem residues (Fig. 8). It has been suggested that effector genes arise from genes used by saprobes to suppress ecological competitors [59, 60]. The steady expression of a series of coregulated candidate effector genes during many months of life on residues, regardless of the changes in the mycoflora present on the residues over time [55], may indicate a need for the fungus to produce a minimal set of molecules involved in ecological competition.

### Heterochromatin-based regulation of expression as a key player in the lifetime coregulation of genes involved in pathogenicity

One of the key findings of our work is that all clusters of genes overexpressed during interaction with the host are significantly associated with localization within a heterochromatin domain during axenic growth. Evidence is accumulating, for many other plant-associated fungi, regardless of their lifestyles and modes of nutrition, that the genes involved in fungus-plant interaction are often present in regions of heterochromatin within the genome (e.g., in [61–63]). Genes highly expressed during infection are often associated with heterochromatin during axenic culture (e.g., in *Z. tritici* or *L. maculans* [4, 37, 64]) and histone modifications (H3K9me3 and / or H3K27me3) have been shown to play a major role in regulating secondary metabolite gene clusters or effector genes (e.g., in *F. graminearum*, *F. fujikuroi*, *Z. tritici*, *Epichloe festucae* [65–69]). In the endophyte *E. festucae* and the pathogen *Z. tritici*, the derepression of putative effector genes or secondary metabolite gene clusters upon infection is associated with an underlying dynamic of histone modifications, which has been experimentally validated [65, 68, 70]. These data suggest that there is a generic chromatin-based control of plant infection mechanisms. We previously showed that, in *L. maculans*, heterochromatin domains (either H3K9me3 or H3K27me3) display significant enrichment in putative effector genes and that all known avirulence genes are associated with H3K9me3 during axenic growth [38]. We found here that the genes included in the eight clusters specifically overexpressed during interaction with the plant relative to axenic growth were also enriched in H3K9me3 and/or H3K27me3 histone modifications in vitro. Although other regulatory mechanisms may be involved, this significant enrichment reinforces the postulate that the underlying condensation state of the chromatin, in the environment of genes sequentially turned on/off during plant colonization, represents an important player in this dynamic. One striking finding of our study is that genes from the various waves of expression are scattered throughout the genome, but are significantly associated with heterochromatin. This association might facilitate the temporal regulation of sets of genes involved in the same process. All the eight expression clusters identified in the lifecycle of *L. maculans* contained genes displaying significant enrichment in H3K27me3 heterochromatin in vitro. This histone modification is an important regulator of development and response to several biotic and abiotic stresses, in various fungal or plant models (e.g., [71, 72]). By contrast, genes associated with biotrophic or biotrophic/asymptomatic life within the plants (cluster 2, Fig. 8) displayed enrichment in H3K9me3 in vitro. The genes in this cluster are located in TE-rich regions of the genome and were found to be more enriched in heterochromatin modifications than the genes of the other clusters. They included the largest number of candidate effector genes, and the largest number of highly coregulated genes (65 genes). In *L. maculans*, the TE-rich regions are comprised of heterochromatin, leading to the silencing of associated genes [37, 38]. The unique association between genes involved in the symptomless spread of the fungus and H3K9me3 suggests that this histone modification is involved in a regulatory mechanism important for the concealment of the fungus from the plants defenses. Conversely, this cluster also contains all the known avirulence genes, providing additional support for the role of H3K9me3 as the primary regulator of expression for genes at the forefront of the battle with the plant, and as a target of choice for the establishment of effector-triggered immunity during co-evolution of the plant and the pathogen.

The genes associated with H3K9me3 in cluster 2 appear to be more strictly coregulated than those in other clusters, in which genes are associated with H3K27me3. Our findings suggest that chromatin remodeling during interaction with the plant is highly dynamic, rather than occurring only once when the fungus infects the plant, as might have been anticipated from analyses of species with a short lifecycle, or from the few days of interaction amenable to laboratory experiments. This combined analysis of transcriptomic and epigenomic data thus supports the notion that the finely tuned temporal progress of fungal infections is underpinned by a highly dynamic phenomenon based on the successive opening and closing of different genomic regions.

## Conclusions

In many organisms, particularly those involved in complex interactions with hosts and the holobiont, many aspects of the biology of the organism cannot be understood through simple biological/epidemiological approaches and cannot be reproduced experimentally. We show here that RNA-Seq approaches, while necessitating complex sampling schemes and complex data processing, are biologically informative and shed light on the various facets of a biological cycle. The data gathered provide us with a more complex view of the fungal life cycle and challenge previous hypotheses and over-simplified views of the interaction of the fungus with its host. Such approaches would also be relevant for complex interactions for which laboratory experiments are difficult or impossible to perform (pure biotrophs, including symbionts, endophytes, organisms with extremely long interactions with perennial plants).

Our data support the notion that fungi harbor genes involved in niche adaptation and that these genes are located in flexible genomic regions, conferring an extreme plasticity of expression and reactivity in the face of changes in the environment in planta. The next challenge will be to visualize and analyze heterochromatin dynamics during in planta colonization.

We were also able to identify stage-specific effectors, including numerous new putative effectors produced during symptomless tissue colonization, or saprophytic growth and survival on residues. The effectors produced during symptomless colonization may, at some stage, be recognized by the plant surveillance machinery and may constitute an invaluable set of potential targets for the identification of novel durable disease resistance genes for breeders.

## Methods

### Plant and fungal materials

Isolate JN2 (v23.1.2) was used in all experiments. Unless otherwise stated, the fungus was cultured on V8-agar medium, and conidia for inoculation tests were produced as previously described [73]. Isolate Nz-T4, which is sexually compatible with JN2, was also used in in vitro crossing experiments. Both isolates were described by Balesdent et al. [74].

Three winter *B. napus* cultivars were used, depending on the experiment: “Darmor-*bzh*,” the reference sequenced cultivar [75], “Darmor” and “Bristol.” “Darmor-*bzh*” is a dwarf isogenic line resulting from the introduction of the dwarf *bzh* gene into “Darmor” [76]. “Darmor-*bzh*” and “Darmor” are characterized by a high level of quantitative resistance in the field, and “Bristol” is moderately susceptible [77].

### Production of biological material for RNA sequencing

#### In vitro culture

The fungus was grown under different in vitro conditions, to mimic as many physiological stages as possible. All replicates used for RNA sequencing were true independent biological replicates. The following physiological conditions were reproduced: mycelial growth on V8-agar medium or in liquid Fries medium, pycnidiospore production on V8-agar medium, pycnidiospore germination in liquid Fries medium, and sexual mating in vitro at three time points (7, 20, and 35 days).

For the mycelial state on solid medium, the fungus was cultivated on V8-agar plates. A plug of an actively growing culture was placed on the center of 9-cm-diameter V8-agar Petri dishes. The plates were sealed with Parafilm and were incubated at 25 °C in the dark. After 7 days of growth the aerial mycelium was collected with a spatula and immediately frozen with liquid nitrogen in an Eppendorf tube. The mycelia from a minimum of three plates were pooled in one sample, and mycelia harvested from two independent experiments were submitted to RNA sequencing.

To produce pycnidiospores, the fungus was cultivated on V8-agar plates without Parafilm and under both white light and near-UV light with a 12-h photoperiod. After 10 days, spores were collected as previously described [73]. The spore suspension from 9 Petri dishes were pooled in 50-mL Falcon tubes and centrifuged for 15 min at 4 °C and 2600*g*. The supernatant was discarded and the spores were suspended in 4 mL of sterile water. One milliliter of this spore suspension was centrifuged similarly in an Eppendorf tube and the pellet was immediately frozen in liquid nitrogen to constitute the “resting spores” sample. For germinating spores, Erlenmeyer containing 20 mL of Fries liquid medium were seeded with the spore suspension at a final concentration of 1.25 × 10^8^ spores mL^− 1^ and submitted to orbital shaking at 150 rotations mn^− 1^. After 24 h at 25 °C, the germinating spore suspension was centrifuged in a Falcon tube for 7 min at 9000*g*, and 4 °C, re-suspended in 1 mL of sterile water for a second centrifugation for 5 min at 9000*g* in an Eppendorf tube. The centrifugate was immediately frozen and stored at − 80 °C to constitute the “germinating spores” sample.

Mycelium was also obtained in liquid cultures in Fries liquid medium as previously established [78] after 7 days of cultivation.

Sexual mating conditions were obtained as previously described [79]. Briefly, plugs of two isolates of the opposite mating types, JN2 and Nz-T4, were deposited side by side on V8 agar plates. After 7 days of cultivation under sporulating conditions, 5 mL of 1.5% water agar were poured over the plates, further sealed with Parafilm and placed at 10–11 °C under backlight with a 12-h photoperiod. Plates with JN2 only were used as a control. Fungal material on the plates was harvested at days 7 (before pouring the agar overlay), 20 and 35 days of cultivation. For each time point, the fungal material (mix of mycelium, pycnidia and maturing pseudothecia) of 10 plates were recovered using a spatula and pooled in one Eppendorf tube, immediately frozen with liquid nitrogen and stored at − 80 °C.

#### Cotyledon inoculation and sampling

The cotyledons of 10-day-old plants of cv Darmor-*bzh* were inoculated with pycnidiospore suspensions, as previously described [80]. Briefly, 48 seeds were sown per tray (4 rows of 12 plants). Ten-microliter droplets of 10^7^ conidia mL^− 1^ suspension were deposited on the center of each half-cotyledon previously punctured with a needle. Plants were maintained in the dark and saturating humidity at room temperature for 48 h then maintained in growth chambers at 16 °C (night)/24 °C (day) with a 16-h light photoperiod. Control plants were mock-inoculated with sterile deionized water. Samples for RNA-Seq were recovered at days 0 (with or without wound), 2, 5, 7, 9, 12, 15, and 17 after inoculation. At each time point, eight cotyledons from eight different plants were randomly selected on the trays. The plant tissues around the inoculation point were cut with a 10-mm disposable punch, and the 16 corresponding samples were pooled together in a sterile Falcon tube, immediately frozen in liquid nitrogen and stored at − 80 °C until extraction. At each time point, two replicates were recovered and the whole experiment was repeated once. At each time point and repeat, mock-inoculated samples were recovered similarly.

#### Petiole inoculation and sampling

The petioles of cvs Darmor-*bzh* and Bristol were inoculated as described by Dutreux et al. [49]. Plants were grown in individual pots and the petioles of the second and third true leaves were inoculated with 8 μL of 10^7^ spores mL^− 1^ pycnidiospore suspensions. Petioles were sampled at 0 (mock), 7, and 14 DPI. At each date, the two inoculated petioles of four plants were cut at their base and placed in Petri dishes. Each petiole was cut 1 cm away from the point of inoculation, and the eight upper fragments obtained were pooled in a 15-mL Falcon tube. The contents of this tube constituted one sample of the upper part of the petiole. All samples were immediately frozen in liquid nitrogen and stored at − 80 °C until extraction. Two independent replicates were recovered at each time point and the whole experiment was repeated.

#### Petiole inoculation and stem sampling

Plants from the three varieties were sown in individual pots (9 × 9 × 9.5 cm) in commercial Falienor substrate (65% Irish blond peat, 20% Baltic black peat, 15% perlite, 2% Danish clay) and grown in a growth chamber (16 h of light at 18 °C at 200 μmol m^− 2^ s^− 1^ and 8 h of dark at 15 °C) for 3 weeks before inoculation. When the plants reached the three-leaf stage, the petiole of the second leaf was cut 0.5 cm from the stem. We applied 10 μL of pycnidiospores (10^7^ spores mL^− 1^) or sterile water (for mock inoculation) to the petiole section. The plants were kept in the dark, under high humidity, for 36 h, and were then transferred to a growth chamber (16 h of light at 20 °C at 200 μmol m^− 2^ s^− 1^ and 8 h of dark at 18 °C). Sub-irrigation was performed using the commercial fertilizer preparation Liquoplant Bleu 0.3% (Plantin, France). Three biological replicates were performed. Each replicate consisted of three blocks of five plants per variety for RNA-Seq sampling. At 14 and 28 DPI, a 0.5 cm-long section of stem was excised from each plant with a razor blade. The stem section was harvested at the level at which the inoculated petiole inserted into the stem, at 14 DPI, and 0.5 cm below this level at 28 DPI. The tissues harvested from each block at each time point (five individual plants) were pooled as a single sample. Harvested tissues were immediately frozen in liquid nitrogen and stored at − 80 °C.

#### Crop residue sampling

In 2012–2013, the susceptible cultivar Alpaga was sown at Grignon, France, where it was subject to natural infection. Disease incidence (% of plants with at least one phoma leaf spot) revealed high levels (80%) of infection in the fall. Disease severity before harvest was estimated on 50 plants using the “G2” rating score as described previously [77]. Only 4% of the plants displayed no stem canker symptom. For 29%, 40%, 22%, and 5% of the plants, the necrotic area represented less than 25%, between 25 and 50%, between 50 and 75%, or more than 75% of the stem section area, respectively, showing variable intensity of plant contamination between plants. After harvest, the stem bases of the plants were removed from the field and kept outside until use, either to reinforce the natural inoculum for the 2013–2014 field assay (see below), or for RNA extraction. For RNA extraction, only plants presenting necrosis on more than 50% of the stem area were used, and stem residues were collected at seven time points after harvest. For one sampling date, two stem residues were pooled to constitute a single sample, and four samples (four replicates) were used per date for RNA sequencing.

#### Stem bases from field sampling

A field experiment was set up at Grignon, France, in the fall of 2013, with two cultivars, Darmor-*bzh* and Bristol. The two cultivars were sown in 10 m × 1.75 m plots in duplicates, at a 60 seeds per m^2^ density. Naturally infected stem residues of the susceptible cv. Alpaga from the previous cropping season were added over the soil the 19/09/2013 to reinforce the natural inoculum. The disease pressure was measured at two dates in autumn (18/10/2013 and 13/15/11/2013). For that, 2 × 50 consecutive plants per micro plot were observed and each plant was classified in one of the following classes: class 0, no visible phoma leaf spot; class 1, less than 5 leaf spots per plant; class 2, more than 5 leaf spots per plant. At the second rating date, the disease incidence (% of infected plants) reached 92.7% for Bristol and 99% for Darmor-*Bzh*. More than 70% and 90% of the plants of Bristol and Darmor-*Bzh*, respectively, displayed more than 5 leaf lesions per plant. Whole plants were collected from this field at 6 time points for RNA extraction (20/11/2013; 18/12/2013; 13/02/2014; 13/03/2014; 4/04/2014; 14/05/2014, 8/07/2014, i.e., 1 week before harvest). For each time point and each cultivar, six plants were collected per micro plot. Plants were washed in the lab with running water and rapidly air dried. Stem section of 1 cm was cut just above the limit between the root and the stem (collar with leaf scares). This piece of stem was then sliced in a sterile Petri dish and the first and last slices were discarded. The central parts of three plants were pooled in one Falcon tube to constitute one sample that was immediately frozen with liquid nitrogen and stored at − 80 °C before RNA extraction. For one given cultivar and time point, 4 samples were thus collected but only three were submitted to RNA sequencing. The stem canker severity (G2 rating, [77]) was calculated on the collected plants starting from the April sampling, and then on 60 plants per plot the 20th June 2014. Only samples from the susceptible cultivar Bristol collected from March to July were kept for RNA-Seq statistical analyses.

#### Young leaves from field sampling

A field experiment was set up at Grignon, France, in the fall of 2017. The cultivar Darmor was sown the 31/08/2017 along with other varieties, in 8 m × 1.75 m plots in triplicates, at a 60 seeds per m^2^ density. Naturally infected stem residues of cv. Alpaga from the previous cropping season were added over the soil the 16/09/2017 at a density of 4 residues per meter square. On 16/11/2017, typical leaf lesions due to *L. maculans* were visible in more than 60% of the plants. Ten leaves per plot with leaf lesions were harvested. In the lab, leaves were washed with running water, rapidly dried between sterile filter papers. Disks of leaf tissues were cut with a sterilized punch (diameter 2 cm), and classified in three categories: disks without any visible symptoms, disks with atypical symptoms potentially attributable to *L. biglobosa*, and disks with typical leaf lesions. Five leaf disks per category were pooled in a 15-mL Falcon tube. All samples were immediately frozen in liquid nitrogen and stored at − 80 °C until extraction. Pooled disks from one plot constitute one replicate, and a similar sampling was performed 2 weeks later. Here, pooled disks from typical leaf lesions only were submitted to RNA-Seq.

#### Infection of cotyledons with ascospores

Stem residues of the cv. Bristol were collected at the end of the 2013–2014 field experiment (see above) and were left outside from July 2014 to March 2015, allowing pseudothecia to differentiate on the stems. Seven-day-old plants of Bristol, grown in 5 × 5 cm pots with 10 plants per pot, were transferred in 50 × 40 cm plastic bowls (24 pots per bowl). Stem residues were rinsed with water, then allowed to dry up again and placed on a metallic grid over the plants (30 residues per bowl). Stem residues were left 24 h over the plants, maintained in the dark with saturated humidity, to allow ascospores to be ejected on the cotyledons. Then the plants were transferred to growth chambers (18–24 °C night-day, 16 h photoperiod as above). After 24 h and 48 h, six cotyledons per pot were randomly cut, pooled in one 15-mL tube, frozen in liquid nitrogen, and stored at − 80 °C. The remaining cotyledons were observed for typical leaf lesions 1 week later. This allowed us to select cotyledons corresponding to pots on which leaf spots, typically due to ascospore due to their rapid development compared to conidia, had developed. Based on this information, cotyledons from 4 neighbor pots on which leaf lesions had developed (i.e., a total of 24 cotyledons) were pooled to produce samples of early stage (24 h and 48 h) of cotyledon infection with ascospores. Two samples were produced at each time point and the experiment was repeated once.

### RNA extraction and sequencing

Cotyledons, leaf disks, and freeze-dried cultures of fungal spores or mycelia were ground with a Retsch MM300 mixer mill in Eppendorf tubes, with one tungsten carbide bead per tube, for 45 s, at 30 oscillations per second. Petioles were ground with a pestle and mortar. Stem bases from the field and stem residues were ground with a Retsch MM300 mixer mill, with zirconium oxide jars and beads, for 40 s, at 30 oscillations per second. Inoculated stem samples were ground in liquid nitrogen, in the presence of steel marbles, with a TissueLyser II (Qiagen).

RNA was extracted with Trizol reagent (Invitrogen, Cergy Pontoise, France), as previously described [18]. RNA-Seq libraries were prepared as previously described [49]. Each library was sequenced with 101 bp paired-end read chemistry, on a HiSeq2000 Illumina sequencer.

### Annotation of genomic data

#### Comparisons of gene annotations between the two published version of the *L. maculans* strain JN3 genome

The reference genome assembly of *L. maculans* isolate v23.1.3 (aka. JN3) was generated in 2007, by Sanger sequencing [26]. Dutreux et al. [49] subsequently generated an improved genome assembly, to achieve assembly at the chromosome scale. We compared this new version of the genome sequence with that published in 2011, using BLAT suite tools [81] (Additional file 16: S4 Text).

#### Functional annotations

Functional annotations were predicted with BLAST2GO [82]. Genes encoding CAZymes were annotated manually, together with de novo prediction with dbCAN tools v3 [83]. PFam domains were annotated with PFamScan software version 1.6 [84]. For discrimination between proteins with and without annotated functions, the BLAST2GO results and PFam annotations were used, but with the terms “hypothetical protein,” “predicted protein,” and “uncharacterized protein” discarded. PKS and NRPS genes were predicted with the SMURF online tool, using default parameters [85].

#### Annotation of genes encoding SSPs

We generated a new repertoire of genes encoding SSPs, by first creating a secretome of predicted secreted proteins with three filters. The predicted secretome contained all the proteins with no more than one transmembrane domain predicted by TMHMM version 2.0 [86], and either a signal peptide predicted by SignalP version 4.1 [87], or an extracellular localization predicted by TargetP version 1.1 [88]. The final SSP repertoire was created by applying a size cutoff of 300 amino acids to the predicted secretome. In parallel, EffectorP version 1.0 [89] was applied to the whole predicted secretome and detected four proteins of more than 300 amino acids in length, which were added to the SSP repertoire.

#### Detection of AT-rich regions

As for the gene models, the new genome version impacted the boundaries of AT/GC isochores previously defined on the first version of the genome assembly [26]. AT-rich and GC-equilibrated regions were thus re-annotated with OcculterCut [90] and post-processed to avoid the detection of regions that were too small. OcculterCut uses a predefined window of 1 kb to analyze AT/GC content, and this can lead to the detection of small GC-rich regions within AT-rich regions in the case of isolated genes. We overcame this problem by defining reliable detected regions (10 kb for AT and 6 kb for GC), which we used as anchors for the merging of regions. Finally, the genome was divided in 553 regions; among them, 290 were AT-rich regions (16.01 Mb, 34.8% genome) and 263 were GC-equilibrated regions (29.96 Mb, 65.2% genome).

### Analyses on genomic data

#### Genomic localization and enrichment in epigenetic marks

For genes in the eight clusters, we performed chi-squared tests to detect enrichment in the associated epigenetic marks (H3K4me2, H3K9me3 or H3K29me3), recovered from Soyer et al. [38], to compare the distribution of marks in the whole set of genes. Enrichments were considered significant for *p* values < 0.05; all analyses were performed in R.

#### Gene ontology enrichment analysis

The Bingo plugin of Cytoscape software v3.5.1 [91] was used for Gene Ontology (GO) enrichment analysis. For each gene, the GO identifier was generated with BLAST2GO software. Significant enrichments in “Molecular Function,” “Biological Process,” and “Cellular Component” were detected with a hypergeometric test, by comparing the proportion of genes identified for a given GO function in the subset of interest to that for the whole set of protein-coding genes in *L. maculans.*

#### Blast

A BLAST alignment was generated with non-redundant protein sequences from the GenPept, Swissprot, PIR, PDF, PDB, and NCBI RefSeq databases. Only the 10 best BLAST hits were selected, with the max_target_seqs: 10 and max_hsps: 10 options.

### Analyses on transcriptomic data

#### Mapping

Raw RNA-Seq reads were mapped with STAR software version 020201 [92]. As *Leptosphaeria biglobosa* isolates could be present in the field samples, we assessed the mapping specificity of the final parameters for the genome of *L. maculans* (Additional files 1 and 2: S1 Text, S1 Table). We chose a number of two base-pair mismatches for a one-step process of mapping onto the concatenated genome of the two species for the final analysis. Based on the maximum intron size within the genome, we allowed for an intron size of 10,000 bp. The other parameters used were as follows: outFilterMultimapNmax: 100; SeedSearchStartLmax: 12; alignSJoverhangMin: 15; alignIntronMin: 10. We then selected the correctly paired reads in BAM files with Samtools v1.6 [93]. Finally, FeaturesCounts version v1.5.1 [94] was used to quantify gene expression for the uniquely mapped and paired reads.

#### Sample correlation analysis

Pairwise Pearson’s correlation analysis was performed on all samples, with the Log2(FPKM + 1)-transformed expression values for the 32 selected sets of conditions. The resulting correlation matrix was clustered with the Ward.D2 method. PCA was performed with the FactoRmineR R package [95] on the scaled Log2(FPKM + 1) expression values for the four groups identified in the correlation matrix.

#### Detection of differentially expressed genes

Differential expression analyses were performed with the EdgeR package v3.20.9 [96]. Genes with > 30 reads in at least one condition were kept for statistical analysis. Samples were normalized by the TMM method. We fitted a negative binomial generalized linear model to the data with the glmFIt function. We then used the glmLRT function to compare gene expression in each of the 22 in planta conditions with that in the 10 in vitro conditions, using appropriate coefficients and contrasts (− 1/10 for the 10 in vitro conditions and + 1 for the tested conditions). We also used coefficients to mask the effects of replicate on cotyledon, petiole and stem samples from controlled conditions. Only genes overexpressed relative to the 10 in vitro conditions with LogFC > 4 and a *p* value < 0.01 were selected.

#### Clustering

For the clustering step, genes with a FPKM > 2 in at least one condition were Log2(FPKM + 1)-transformed and scaled. The whole 1207 gene set overexpressed in at least one set of conditions in planta was extracted from this transformed count table, and the self-organizing map method of the Kohonen R package v3.0.8 [97] was used to classify those genes into eight clusters, with 200 iterations.

#### Detection of co-expressed genes

For each cluster, the mean scaled Log2(FPKM + 1) value for SSP gene expression was calculated and used as a reference for an analysis of linear regression against the scaled Log2(FPKM + 1) expression of all genes. Genes with an *R*-squared value > 0.80 and an adjusted *p* value < 0.05 were considered to be highly correlated.

## Supplementary Information

**Additional file 1: S1 Text.** Characteristics of biological and RNA samples.

**Additional file 2: Table S1.** Results of the mapping parameters tested for distinguishing between reads from *L. maculans* and *L. biglobosa. (PDF 37 kb)*

**Additional file 3: Table S2.** Mapping results for the 102 samples subjected to RNA sequencing on the *L. maculans* and *L. biglobosa* genomes.

**Additional file 4: S2 Text.** Reproducibility between replicates.

**Additional file 5: Fig. S1.** Principal component analyses (PCA) of RNA-Seq replicates. Genes with an FPKM count > 2 in at least two sets of conditions were selected and the Log_2_(FPKM+ 1) value of each replicate was used as an input for PCA. PCA was performed separately for each group (GP1 to GP4) identified in the correlation analysis (Fig. 2), and the results are shown in (A) to (D). Samples are named according to Fig. 1 and the last number indicates the replicate number. The two axes represent the first and second principal components. In each PCA, a color is attributed to each independent set of in vitro or in planta time-course experiment. The ellipses represent the variability of each group, with a confidence interval of 95%.

**Additional file 6: S3 Text.** Generation of a new repertoire of small secreted proteins considered as candidate effectors.

**Additional file 7: Fig. S2.** Detection of Gene Ontology enrichment (“Biological Process” category) in each of the eight clusters containing the 1207 *Leptosphaeria maculans* genes overexpressed in at least one set of conditions in planta relative to the 10 sets of in vitro conditions. For each cluster, enrichment in a Biological Process category was assessed in a hypergeometric test, with the Cytoscape tool Bingo [89]. The *y*-axis indicates the overrepresented Biological Process terms. The *x*-axis represents the resulting -Log_10_(corrected *p*-value) of the enrichment test. The numbers in the boxes indicate the number of genes assigned to the corresponding Biological Processes in the cluster (left) and the total number of genes associated to this Biological Process for the whole gene set (right). No significant enrichment in Biological Processes was found in clusters 2, 3 and 5.

**Additional file 8: Fig. S3.** Detection of Gene Ontology enrichment (“Molecular Function” category) in each of the eight clusters containing the 1207 *Leptosphaeria maculans* genes overexpressed in at least one set of in planta conditions relative to the 10 sets of in vitro conditions. For each cluster, enrichment in a particular Molecular Function category was assessed in a hypergeometric test, with the Cytoscape tool Bingo [89]. The *y*-axis indicates the overrepresented Molecular Function terms. The *x*-axis represents the -Log_10_(corrected *p*-value) of enrichment for each Molecular Function. The numbers in the boxes indicate the number of genes assigned to the corresponding Molecular Function in the clusters (left) and the total number of genes associated to this Molecular Function term in the whole gene set (right). The ‘small secreted protein’ category, which is not present in the GO database, was added to the statistical tests.

**Additional file 9: Fig. S4.** Detection of Gene Ontology enrichment (“Cellular Component” category) in each of the eight clusters containing the 1207 *Leptosphaeria maculans* genes overexpressed in at least one set of conditions in planta relative to the 10 sets of conditions in vitro*.* For each cluster, we assessed the enrichment in a particular Cellular Component category in a hypergeometric test with the Cytoscape tool Bingo [89]. The *y*-axis indicates the overrepresented Cellular Component terms. The *x*-axis represents the -Log_10_(corrected *p*-value) of the enrichment for each corresponding Cellular Component. The numbers in the boxes indicate the number of genes assigned to the corresponding Cellular Component in the cluster (left) and the total number of genes associated to this Cellular Component term in the whole gene set (right). No significant enrichment in Cellular Components was found in clusters 2, 6 and 8.

**Additional file 10: Fig. S5.** Proportion of different classes of CAZymes and their substrates in the eight gene clusters. (A) The proportion of the five CAZyme classes and one subclass of CAZymes with auxiliary activities (LPMO: lytic polysaccharide monooxygenase) within the clusters (cluster 1 to 8) or the whole gene set of *Leptosphaeria maculans* (*N*, number of genes in each cluster). (B) The proportion (*y*-axis: from 0 to 1) of the identified CAZyme substrates relative to the total number of CAZymes in each cluster (N).

**Additional file 11: Fig. S6.** Proportion of proteins of each cluster conserved in other fungal species. For each protein sequence in each cluster, the top ten BLAST alignments on the NCBI non-redundant protein database were extracted (e-value < 1.10^− 5^). The proportion of protein sequences aligned with those of at least one fungal species is shown in gray and the proportion of proteins aligned only with *L. maculans* proteins is shown in black. (N) is the total number of *L. maculans* genes in the cluster.

**Additional file 12: Fig. S7.** Proportion of BLAST matches among four genera of phytopathogenic fungi. For each *L. maculans* gene in each cluster, the top ten of BLAST alignments against the NCBI non-redundant protein database were extracted. BLAST matches on species belonging to four fungal genera *Colletotrichum* (in orange, represented by 13 species), *Fusarium* (in cyan, represented by 12 species), *Zymoseptoria* (in gray, represented by one species, *Zymoseptoria tritici*) and *Botrytis* (in pink, represented by one species, *Botrytis cinerea*) were extracted. All proteins without predicted function were discarded. The bars represent the proportion of homologous proteins with an annotated function depending on the genus selected. (N) is the total number of proteins with BLAST hits and with a predicted function, all genera included.

**Additional file 13: Table S3.** Detection and cluster distribution of PKS and NRPS genes among the 1207 *Leptosphaeria maculans* genes upregulated in planta*. (PDF 31 kb)*

**Additional file 14: Fig. S8.** Details of the three *Leptosphaeria maculans* secondary metabolite biosynthesis gene clusters upregulated during rapeseed infection. The genomic regions and scaffolds are represented by the gray bars, with the coordinates indicated beneath them. The red bars correspond to AT-rich regions. The annotations of the genes involved in the abscisic acid (ABA) or sirodesmin biosynthesis are indicated. One secondary metabolite (SM) biosynthesis gene cluster was annotated de novo and its borders were delimited by the co-expression of 10 neighboring genes, with one gene encoding an NRPS-like protein. The genes in green represent the genes annotated in the SM cluster in previous studies but not found to be overexpressed during rapeseed infection. The genes in black represent the genes found to be overexpressed during rapeseed infection and present in the cluster. The gene names are indicated for the ABA [27] and sirodesmin biosynthesis cluster [44]. For the de novo annotated cluster, the functional annotations predicted by BLAST2GO are indicated (MT: methyltransferase, IM: integral membrane, TF: transcription factor, MP: maltose permease, CytP450: cytochrome P450, Na: no predicted function, MFS, major facilitator superfamily).

**Additional file 15: Table S4.** Description and cluster assignment of the 52 genes for which molecular studies have been performed.

**Additional file 16: S4 Text.** Comparison between the v1 and v2 versions of gene model annotations.

**Additional file 17: Fig. S9.** Characteristics of the small secreted proteins (SSP) predicted from the *Leptosphaeria maculans* protein set. (A) Protein features used to predict the SSP repertoire. The Venn diagram contains all proteins with no more than one transmembrane domain. The numbers of proteins with a predicted signal peptide (SP, predicted by SignalP), a predicted extracellular localization (EL: predicted by TargetP), a size of less than 300 amino acids (300 aa), and predicted to be SSPs by the EffectorP tool (EP) are indicated. The black circle includes all proteins comprising the SSP repertoire. (B) Relationship between functional annotation, percentage of cysteine residues and protein size of the 1892 proteins of the *L. maculans* secretome. The predicted secretome includes all proteins with no more than one transmembrane domain, with a predicted signal peptide or a predicted extracellular localization. The percentage of cysteine residues was calculated (gray dot) and the trend line obtained with the GAM function (generalized additive models with integrated smoothness estimation) is plotted to indicate the change of the percent cysteine residues with protein size. The histogram represents the percentage of genes with and without a predicted function, in blue and red, respectively. The dotted line represents the protein size cutoff (300 amino acids) applied to the secretome.

**Additional file 18: Table S5.** Characteristics of the 1070 genes encoding small secreted proteins (SSP).

**Additional file 19: Table S6.** Comparison of protein features and genomic localization between the small secreted protein (SSP) repertoire predicted from v2 genome annotation relative to the whole gene set with those obtained for the previously predicted SSP repertoire.

**Additional file 20: Fig. S10.** Expression of the 271 small secreted protein-encoding genes upregulated in at least one of the 22 sets of conditions in planta*.* The scaled Log_2_(FPKM+ 1) expression values for the 271 SSP genes upregulated in at least one set of conditions in planta condition are shown, grouped according to cluster assignment. The distribution and expression of the nine avirulence effector genes (*AvrLm)*, and the eight late candidate effector genes [14] are highlighted. The mean expression level of the SSP genes in each of the eight clusters is plotted (black bold curve) and the total number of SSP genes in each cluster is indicated. Three sample features are described: (i) the experimental conditions: IPF, in planta field conditions; IPC, in planta controlled conditions; RF, residues in field conditions, (ii) the type of plant tissue sampled and (iii) the sampling time points (DPI, days post-inoculation; MPS, months post-sowing; MPH, months post harvest).

**Additional file 21: S5 Text.** Genes expressed when ascospores colonize the plant tissues or when the fungus is dormant in the stem.

**Additional file 22: Table S7.** Genes with more than 10 reads in (A) at least one of the four replicates of samples at 24 h or 48 h after ascospore ejection on cotyledons (B) at least one of the nine replicates from the three samples of stem base tissues at 2, 3 or 5 months post sowing.

**Additional file 23: Table S8.** Conservation of the protein sequences encoded by highly co-expressed SSP genes.

## Data Availability

Accession numbers of RNA-Seq data are available in supplementary Table S2. Other data are available in the Supplementary files.
