# Supplementary material for: Large-scale transcriptomics to dissect 2 years of the life of a fungal phytopathogen interacting with its host plant
Source: BMC Biol. 2021 Mar 23;19:55. doi: 10.1186/s12915-021-00989-3 (PMC7986464; doi:10.1186/s12915-021-00989-3)
Supplement: Supplementary file 2 — Additional file 2: Table S1. Results of the mapping parameters tested for distinguishing between reads from L. maculans and L. biglobosa. (PDF 37 kb) [file 12915_2021_989_MOESM2_ESM.pdf]

S1 Table. Results of the mapping parameters tested for distinguishing between reads from *L. maculans* and *L. biglobosa.*

[a.] Reads of the two samples from *in vitro* cultures of either *L. maculans* (isolate JN2) or *L. biglobosa* (isolate G12-14) were mapped independently on the genomes of *L. maculans* (isolate JN3) and *L. biglobosa* isolate G12-14)

[b.] Reads of the two samples from *in vitro* cultures of either *L. maculans* (isolate JN2) or *L. biglobosa* (isolate G12-14) were both mapped on one artificial genome created by the concatenation of the *L. maculans* (isolate JN3) and *L. biglobosa* (isolate G12-14) genomes.

**Reference mapping genome**	**RNAseq samples used**	**Number of mismatch allowed**	**% of reads mapped on *L. maculans* (JN3) genome**	**% of reads mapped on *L. biglobosa* (G12-14) genome**
[a.] JN3 or G12-14 genomes	*In vitro* culture of *L. maculans* (JN2)	0	96.62	0.46
		1	97.27	0.97
		2	97.42	1.63
	In vitro culture of *L. biglobosa* (G12-14)	0	0.76	95.13
		1	1.54	95.59
		2	2.66	95.69
[b.] JN3 and G12-14 concatenated genomes	*In vitro* culture of *L. maculans* (JN2)	0	96.7	0.04
		1	97.32	0.06
		2	97.45	0.08
	In vitro culture of *L. biglobosa* (G12-14)	0	0.10	95.15
		1	0.11	95.58
		2	0.12	95.67
