# Supplementary material for: Large-scale transcriptomics to dissect 2 years of the life of a fungal phytopathogen interacting with its host plant
Source: BMC Biol. 2021 Mar 23;19:55. doi: 10.1186/s12915-021-00989-3 (PMC7986464; doi:10.1186/s12915-021-00989-3)
Supplement: Supplementary file 4 — Additional file 4: S2 Text. Reproducibility between replicates. [file 12915_2021_989_MOESM4_ESM.pdf]

S2 Text. Reproducibility between replicates.

Variability between replicates was analysed with a PCA approach and indicated consistency between replicates in most of the samples (Additional file 5: S1 Fig). The highest variability was detected between replicates from asymptomatic field-infected stem bases (Additional file 5: S1A Fig) or crop residues (Additional file 5: S1C Fig) probably linked to numerous environmental factors causing transcriptomic variability from one replicate to another or variable intensity of fungal colonization from one plant to the other. One series of *in vitro* growth conditions, those promoting differentiation of pycnidia and/or pseudothecia, also showed some variability between replicates (Additional file 5: S1D Fig) that may reflect lack of synchronicity from one sample to the other in the differentiation processes.
