# Supplementary figures and images for: Large-scale transcriptomics to dissect 2 years of the life of a fungal phytopathogen interacting with its host plant
Source: BMC Biol. 2021 Mar 23;19:55. doi: 10.1186/s12915-021-00989-3 (PMC7986464; doi:10.1186/s12915-021-00989-3)

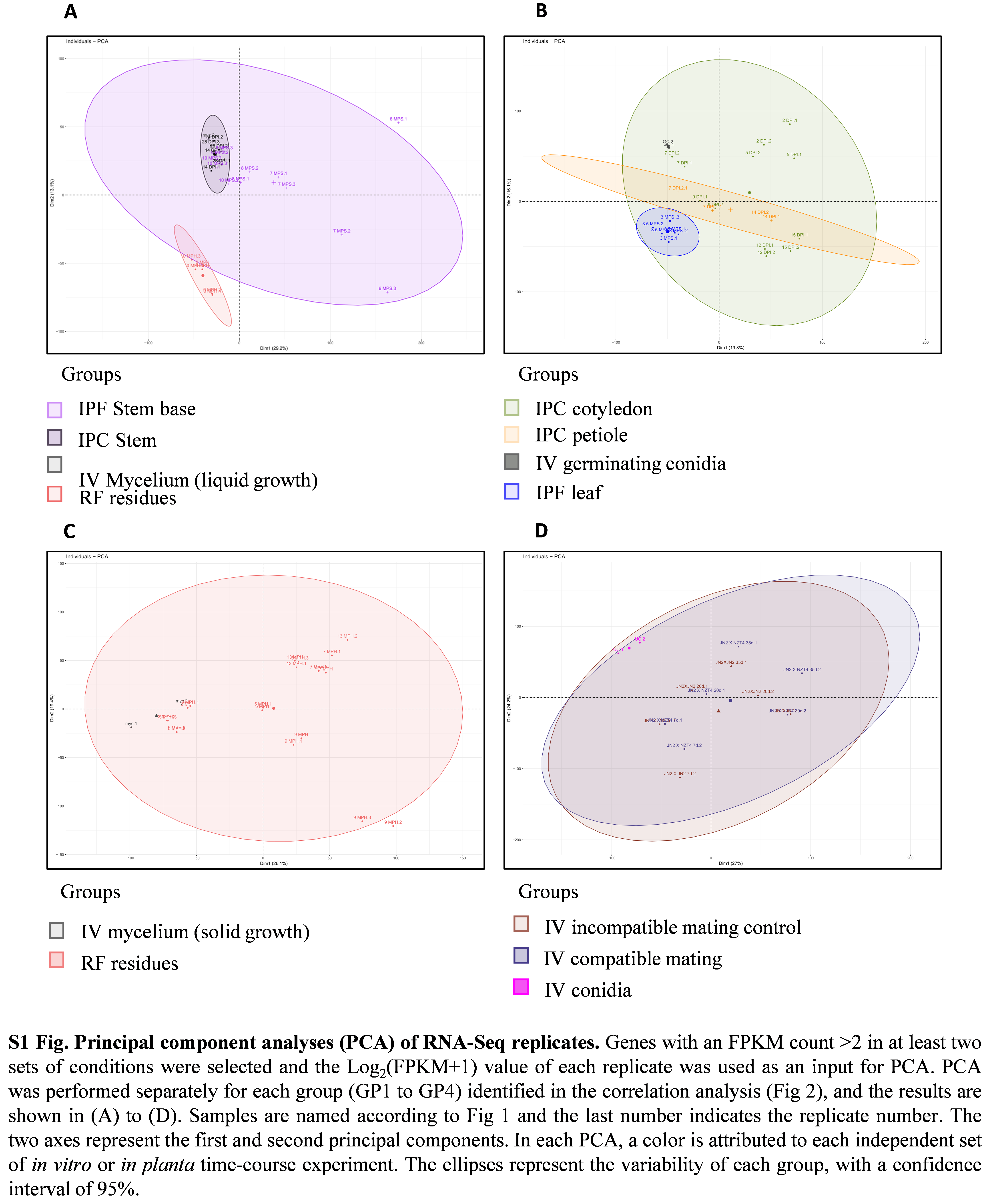

Supplement: Supplementary file 5 — Additional file 5: Fig. S1. Principal component analyses (PCA) of RNA-Seq replicates. Genes with an FPKM count > 2 in at least two sets of conditions were selected and the Log2(FPKM+ 1) value of each replicate was used as an input for PCA. PCA was performed separately for each group (GP1 to GP4) identified in the correlation analysis (Fig. 2), and the results are shown in (A) to (D). Samples are named according to Fig. 1 and the last number indicates the replicate number. The two axes represent the first and second principal components. In each PCA, a color is attributed to each independent set of in vitro or in planta time-course experiment. The ellipses represent the variability of each group, with a confidence interval of 95%. [file 12915_2021_989_MOESM5_ESM.tif]

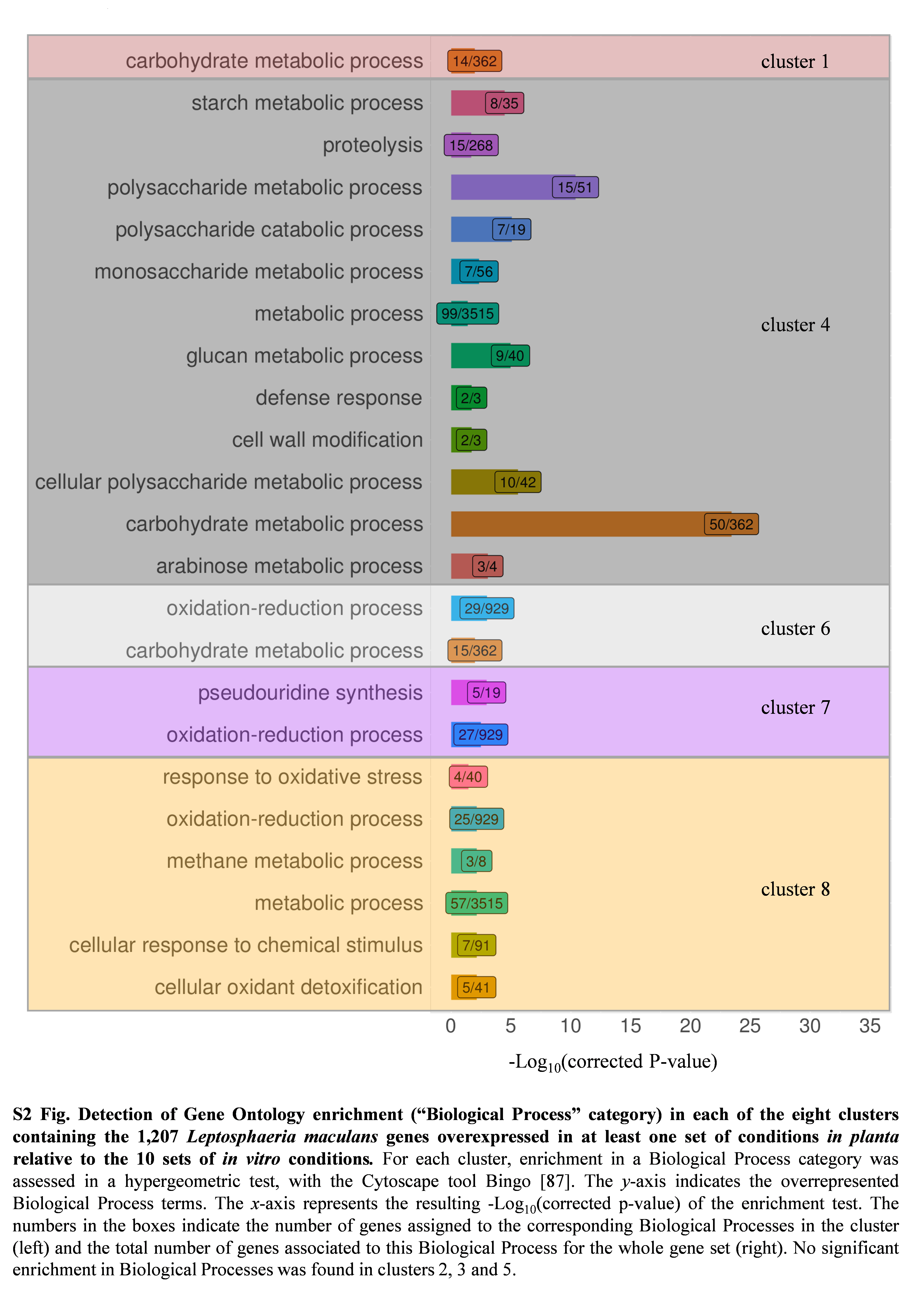

Supplement: Supplementary file 7 — Additional file 7: Fig. S2. Detection of Gene Ontology enrichment (“Biological Process” category) in each of the eight clusters containing the 1207 Leptosphaeria maculans genes overexpressed in at least one set of conditions in planta relative to the 10 sets of in vitro conditions. For each cluster, enrichment in a Biological Process category was assessed in a hypergeometric test, with the Cytoscape tool Bingo [89]. The y-axis indicates the overrepresented Biological Process terms. The x-axis represents the resulting -Log10(corrected p-value) of the enrichment test. The numbers in the boxes indicate the number of genes assigned to the corresponding Biological Processes in the cluster (left) and the total number of genes associated to this Biological Process for the whole gene set (right). No significant enrichment in Biological Processes was found in clusters 2, 3 and 5. [file 12915_2021_989_MOESM7_ESM.tif]

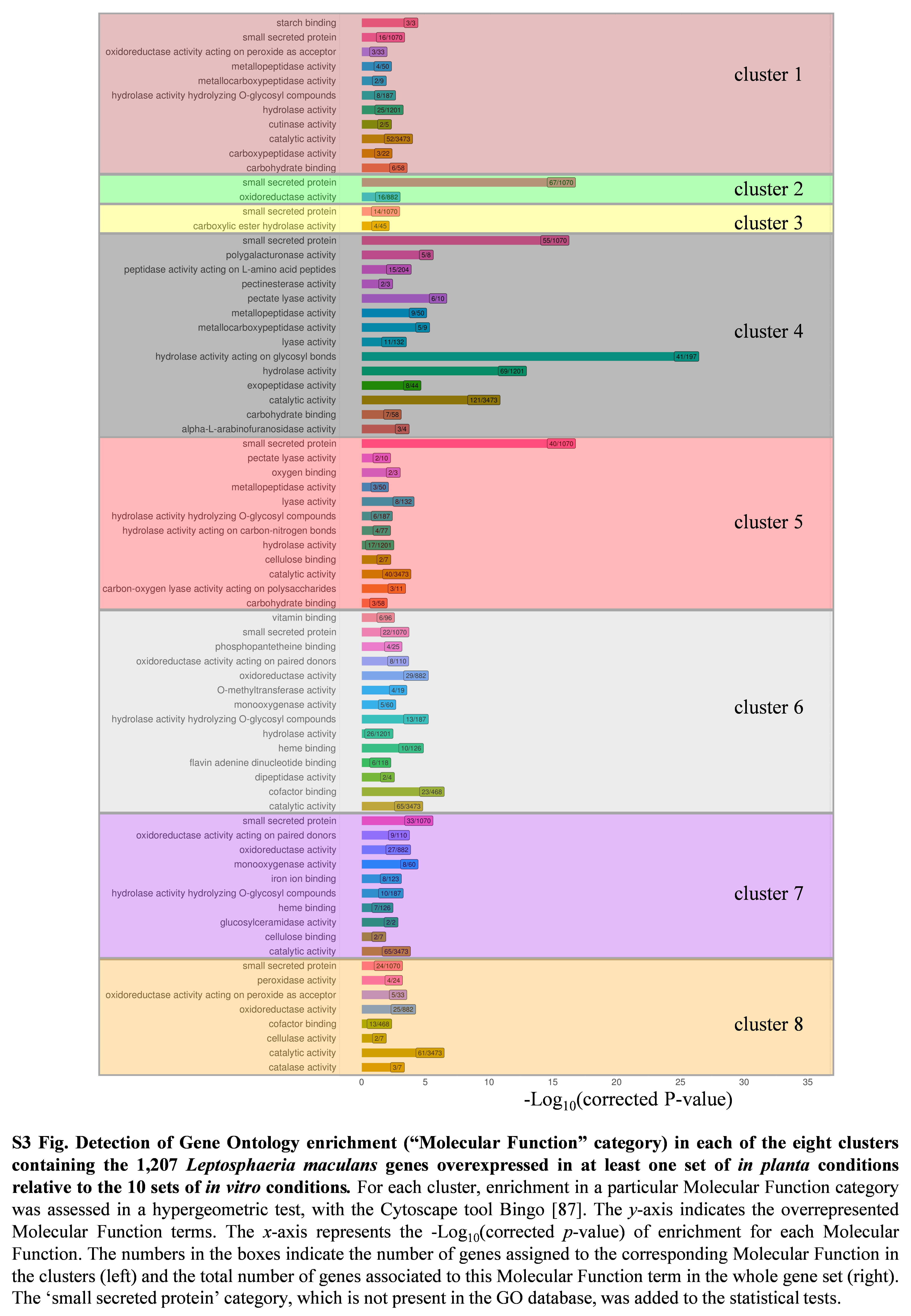

Supplement: Supplementary file 8 — Additional file 8: Fig. S3. Detection of Gene Ontology enrichment (“Molecular Function” category) in each of the eight clusters containing the 1207 Leptosphaeria maculans genes overexpressed in at least one set of in planta conditions relative to the 10 sets of in vitro conditions. For each cluster, enrichment in a particular Molecular Function category was assessed in a hypergeometric test, with the Cytoscape tool Bingo [89]. The y-axis indicates the overrepresented Molecular Function terms. The x-axis represents the -Log10(corrected p-value) of enrichment for each Molecular Function. The numbers in the boxes indicate the number of genes assigned to the corresponding Molecular Function in the clusters (left) and the total number of genes associated to this Molecular Function term in the whole gene set (right). The ‘small secreted protein’ category, which is not present in the GO database, was added to the statistical tests. [file 12915_2021_989_MOESM8_ESM.tif]

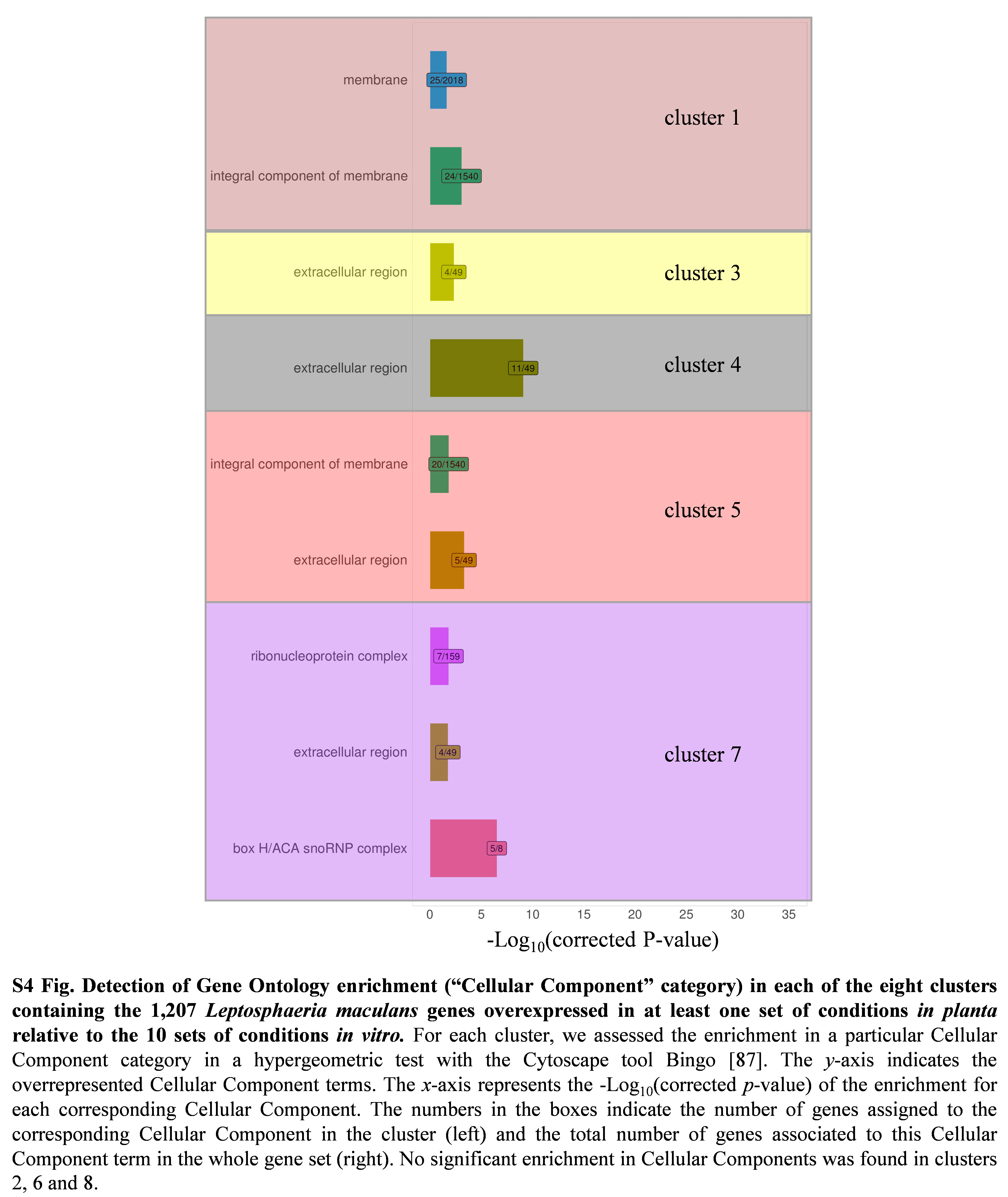

Supplement: Supplementary file 9 — Additional file 9: Fig. S4. Detection of Gene Ontology enrichment (“Cellular Component” category) in each of the eight clusters containing the 1207 Leptosphaeria maculans genes overexpressed in at least one set of conditions in planta relative to the 10 sets of conditions in vitro. For each cluster, we assessed the enrichment in a particular Cellular Component category in a hypergeometric test with the Cytoscape tool Bingo [89]. The y-axis indicates the overrepresented Cellular Component terms. The x-axis represents the -Log10(corrected p-value) of the enrichment for each corresponding Cellular Component. The numbers in the boxes indicate the number of genes assigned to the corresponding Cellular Component in the cluster (left) and the total number of genes associated to this Cellular Component term in the whole gene set (right). No significant enrichment in Cellular Components was found in clusters 2, 6 and 8. [file 12915_2021_989_MOESM9_ESM.tif]

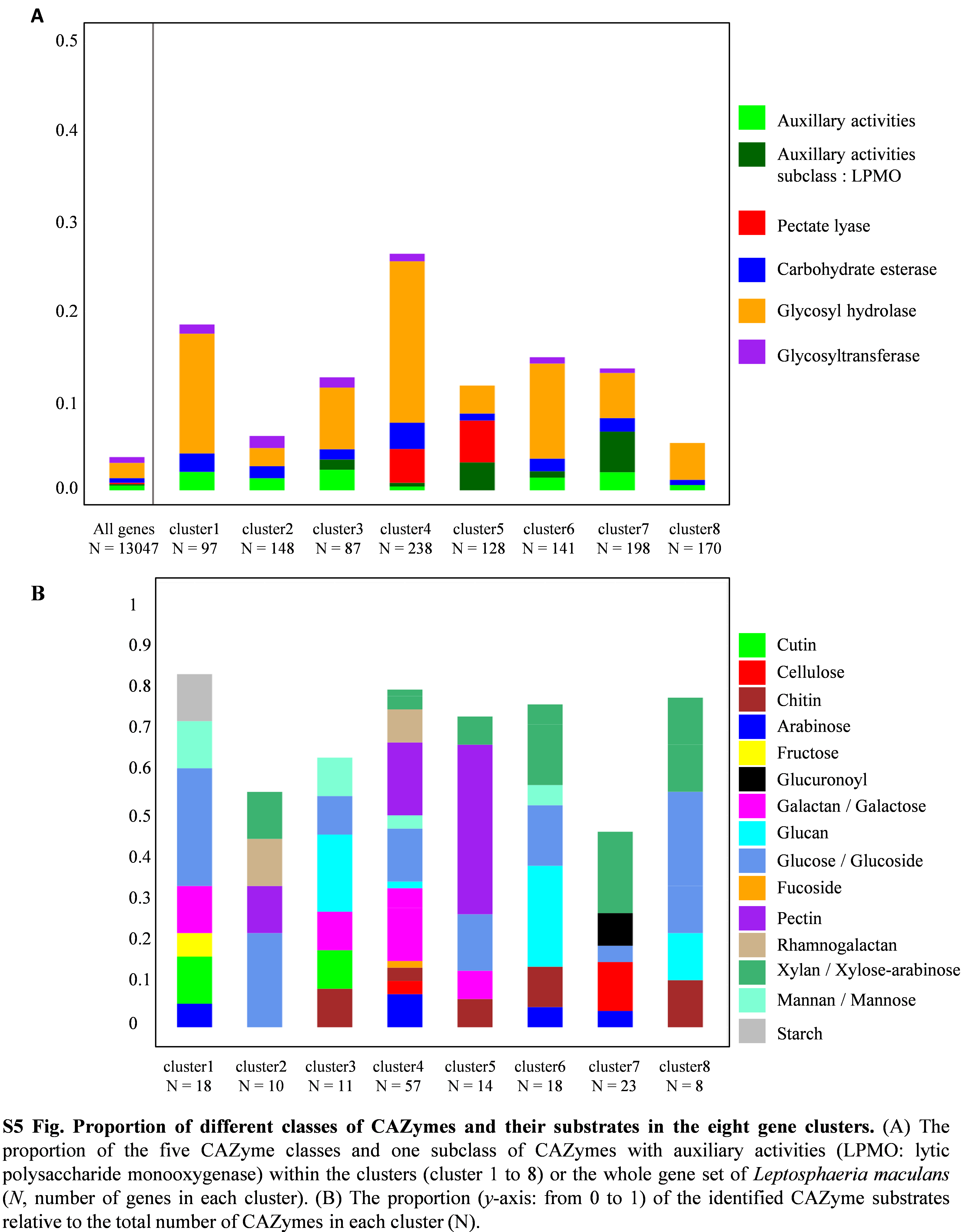

Supplement: Supplementary file 10 — Additional file 10: Fig. S5. Proportion of different classes of CAZymes and their substrates in the eight gene clusters. (A) The proportion of the five CAZyme classes and one subclass of CAZymes with auxiliary activities (LPMO: lytic polysaccharide monooxygenase) within the clusters (cluster 1 to 8) or the whole gene set of Leptosphaeria maculans (N, number of genes in each cluster). (B) The proportion (y-axis: from 0 to 1) of the identified CAZyme substrates relative to the total number of CAZymes in each cluster (N). [file 12915_2021_989_MOESM10_ESM.tif]

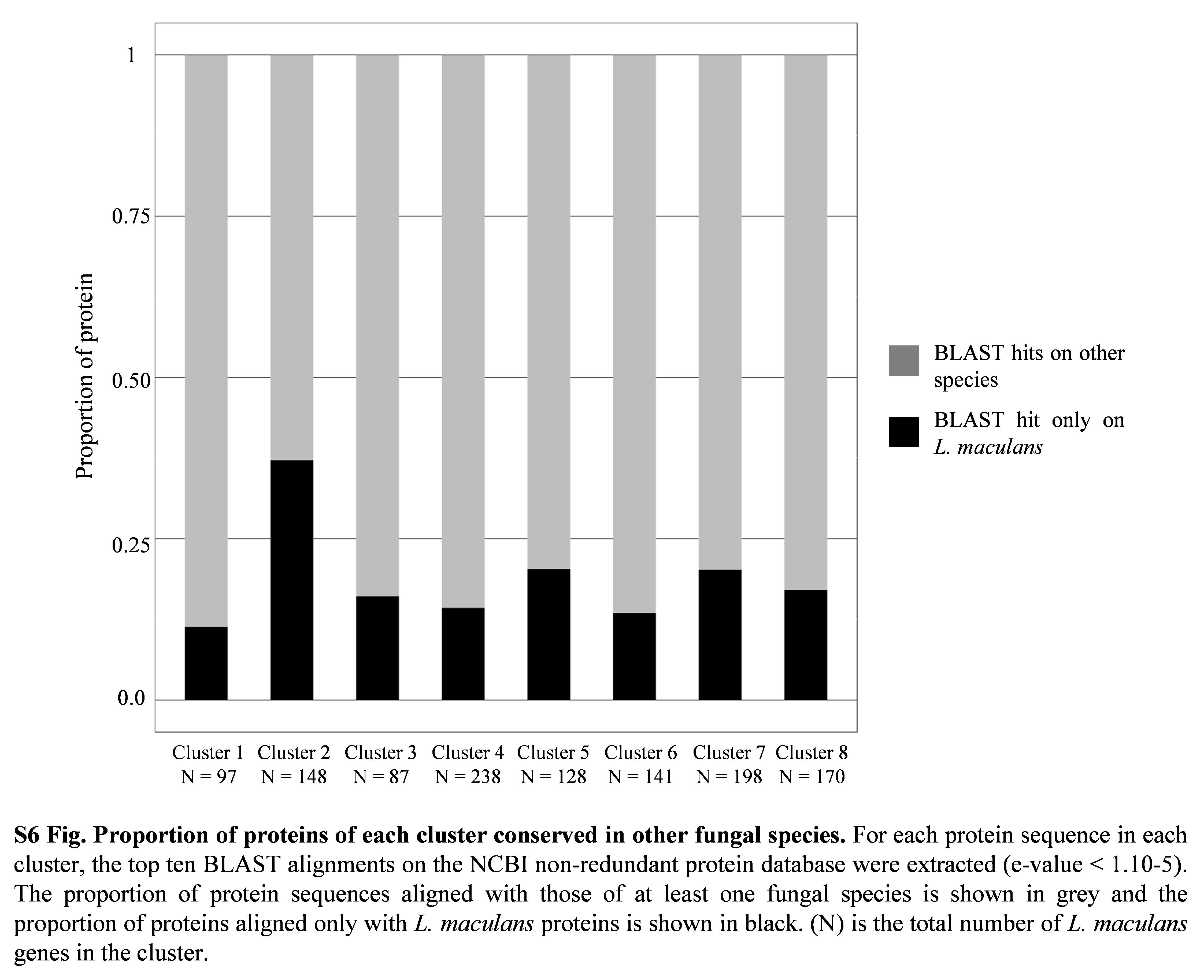

Supplement: Supplementary file 11 — Additional file 11: Fig. S6. Proportion of proteins of each cluster conserved in other fungal species. For each protein sequence in each cluster, the top ten BLAST alignments on the NCBI non-redundant protein database were extracted (e-value < 1.10− 5). The proportion of protein sequences aligned with those of at least one fungal species is shown in gray and the proportion of proteins aligned only with L. maculans proteins is shown in black. (N) is the total number of L. maculans genes in the cluster. [file 12915_2021_989_MOESM11_ESM.tif]

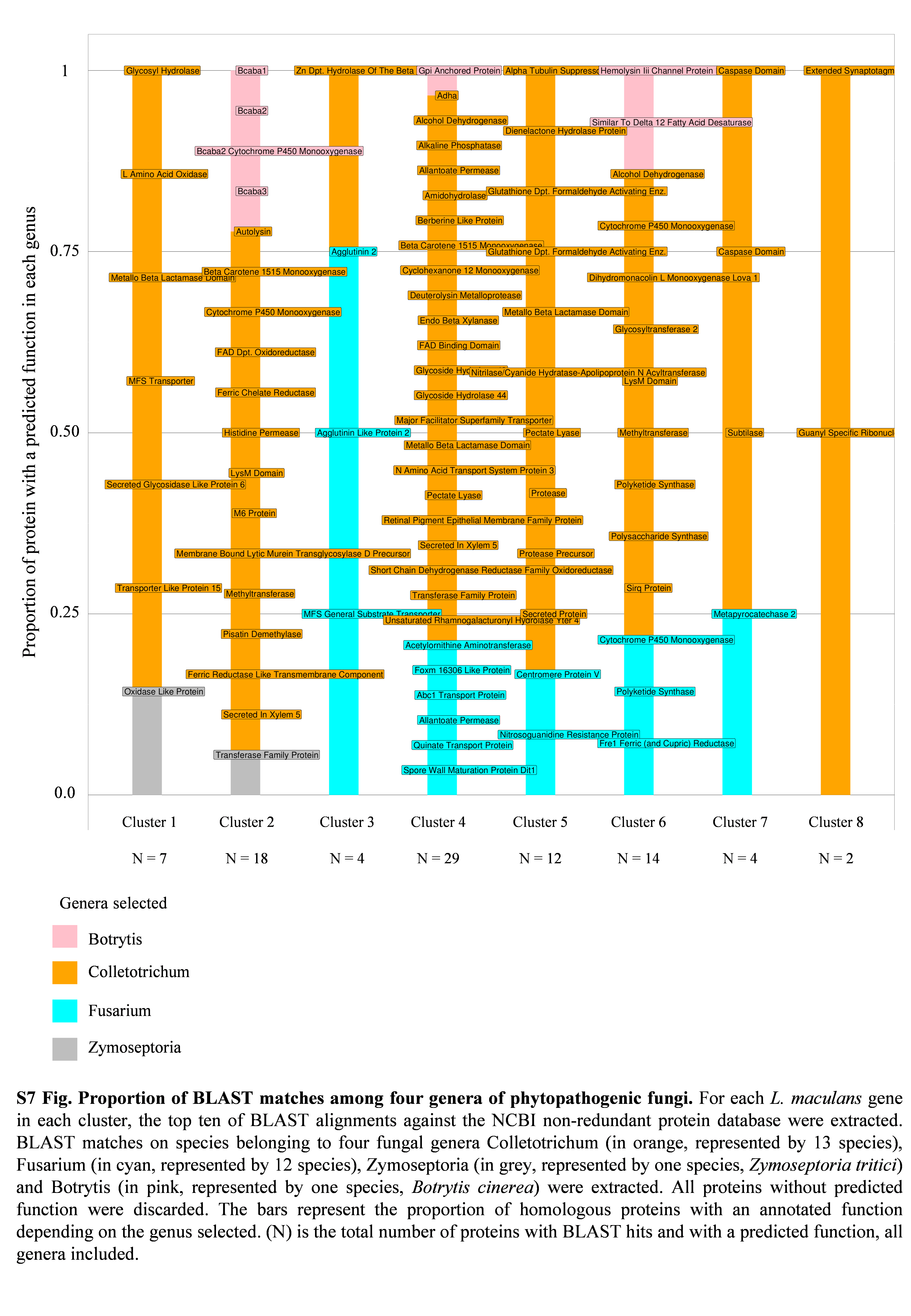

Supplement: Supplementary file 12 — Additional file 12: Fig. S7. Proportion of BLAST matches among four genera of phytopathogenic fungi. For each L. maculans gene in each cluster, the top ten of BLAST alignments against the NCBI non-redundant protein database were extracted. BLAST matches on species belonging to four fungal genera Colletotrichum (in orange, represented by 13 species), Fusarium (in cyan, represented by 12 species), Zymoseptoria (in gray, represented by one species, Zymoseptoria tritici) and Botrytis (in pink, represented by one species, Botrytis cinerea) were extracted. All proteins without predicted function were discarded. The bars represent the proportion of homologous proteins with an annotated function depending on the genus selected. (N) is the total number of proteins with BLAST hits and with a predicted function, all genera included. [file 12915_2021_989_MOESM12_ESM.tif]

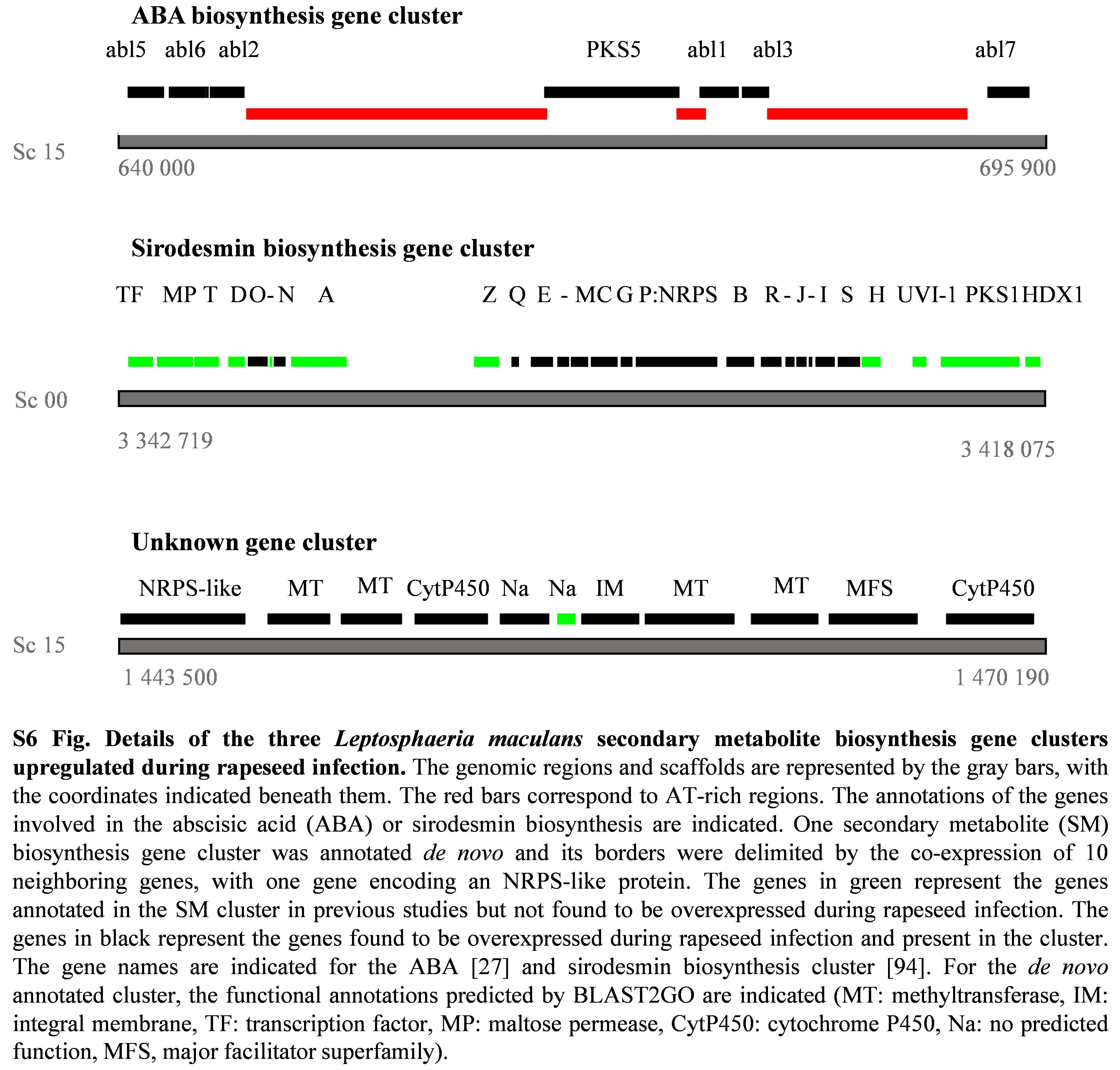

Supplement: Supplementary file 14 — Additional file 14: Fig. S8. Details of the three Leptosphaeria maculans secondary metabolite biosynthesis gene clusters upregulated during rapeseed infection. The genomic regions and scaffolds are represented by the gray bars, with the coordinates indicated beneath them. The red bars correspond to AT-rich regions. The annotations of the genes involved in the abscisic acid (ABA) or sirodesmin biosynthesis are indicated. One secondary metabolite (SM) biosynthesis gene cluster was annotated de novo and its borders were delimited by the co-expression of 10 neighboring genes, with one gene encoding an NRPS-like protein. The genes in green represent the genes annotated in the SM cluster in previous studies but not found to be overexpressed during rapeseed infection. The genes in black represent the genes found to be overexpressed during rapeseed infection and present in the cluster. The gene names are indicated for the ABA [27] and sirodesmin biosynthesis cluster [44]. For the de novo annotated cluster, the functional annotations predicted by BLAST2GO are indicated (MT: methyltransferase, IM: integral membrane, TF: transcription factor, MP: maltose permease, CytP450: cytochrome P450, Na: no predicted function, MFS, major facilitator superfamily). [file 12915_2021_989_MOESM14_ESM.tif]

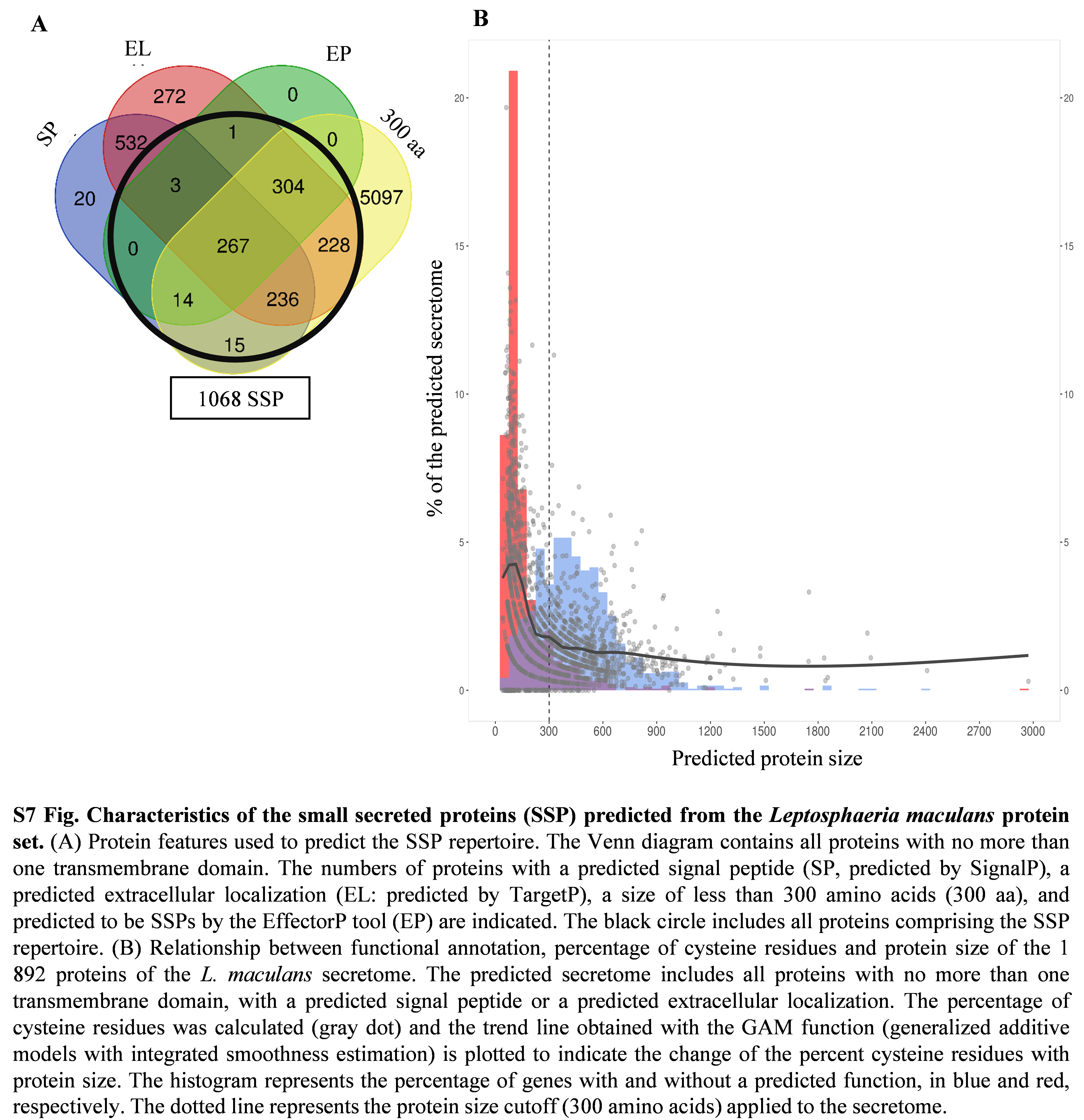

Supplement: Supplementary file 17 — Additional file 17: Fig. S9. Characteristics of the small secreted proteins (SSP) predicted from the Leptosphaeria maculans protein set. (A) Protein features used to predict the SSP repertoire. The Venn diagram contains all proteins with no more than one transmembrane domain. The numbers of proteins with a predicted signal peptide (SP, predicted by SignalP), a predicted extracellular localization (EL: predicted by TargetP), a size of less than 300 amino acids (300 aa), and predicted to be SSPs by the EffectorP tool (EP) are indicated. The black circle includes all proteins comprising the SSP repertoire. (B) Relationship between functional annotation, percentage of cysteine residues and protein size of the 1892 proteins of the L. maculans secretome. The predicted secretome includes all proteins with no more than one transmembrane domain, with a predicted signal peptide or a predicted extracellular localization. The percentage of cysteine residues was calculated (gray dot) and the trend line obtained with the GAM function (generalized additive models with integrated smoothness estimation) is plotted to indicate the change of the percent cysteine residues with protein size. The histogram represents the percentage of genes with and without a predicted function, in blue and red, respectively. The dotted line represents the protein size cutoff (300 amino acids) applied to the secretome. [file 12915_2021_989_MOESM17_ESM.tif]

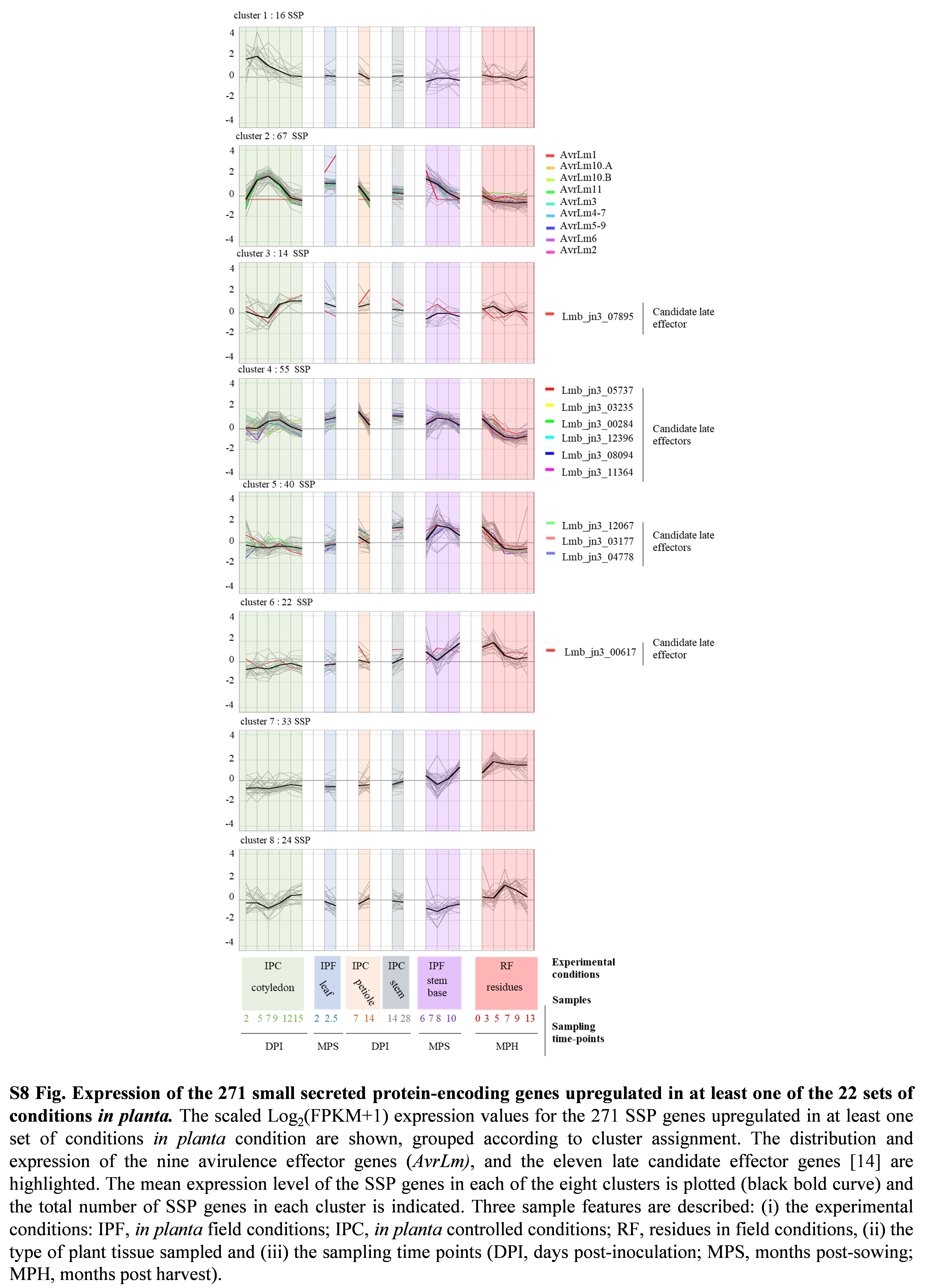

Supplement: Supplementary file 20 — Additional file 20: Fig. S10. Expression of the 271 small secreted protein-encoding genes upregulated in at least one of the 22 sets of conditions in planta. The scaled Log2(FPKM+ 1) expression values for the 271 SSP genes upregulated in at least one set of conditions in planta condition are shown, grouped according to cluster assignment. The distribution and expression of the nine avirulence effector genes (AvrLm), and the eight late candidate effector genes [14] are highlighted. The mean expression level of the SSP genes in each of the eight clusters is plotted (black bold curve) and the total number of SSP genes in each cluster is indicated. Three sample features are described: (i) the experimental conditions: IPF, in planta field conditions; IPC, in planta controlled conditions; RF, residues in field conditions, (ii) the type of plant tissue sampled and (iii) the sampling time points (DPI, days post-inoculation; MPS, months post-sowing; MPH, months post harvest). [file 12915_2021_989_MOESM20_ESM.tif]
