# Supplementary material for: Large-scale transcriptomics to dissect 2 years of the life of a fungal phytopathogen interacting with its host plant
Source: BMC Biol. 2021 Mar 23;19:55. doi: 10.1186/s12915-021-00989-3 (PMC7986464; doi:10.1186/s12915-021-00989-3)
Supplement: Supplementary file 22 — Additional file 22: Table S7. Genes with more than 10 reads in (A) at least one of the four replicates of samples at 24 h or 48 h after ascospore ejection on cotyledons (B) at least one of the nine replicates from the three samples of stem base tissues at 2, 3 or 5 months post sowing. [file 12915_2021_989_MOESM22_ESM.pdf]

S7 Table. Genes with more than 10 reads in (A) at least one of the four replicates of samples at 24 h or 48 h after ascospore ejection on cotyledons (B) at least one of the nine replicates from the three samples of stem base tissues at 2, 3 or 5 months post sowing.

[a] Total number of genes with a count > 10 reads in at least one of the replicates at 24h or 48h post infection by ascospores

[b] No of genes absent from the set of 1,207 genes over-expressed in one of the 22 analyzable conditions compared to the ten *in vitro* conditions

[c] No of genes present in the set of 1,207 genes over-expressed in one of the 22 analyzable conditions compared to the ten *in vitro* conditions, and their cluster assignation (cluster 1 to cluster 8).

A

**Gene categories**		**Non-SSP**	**SSP**
Total number of genes with a count > 10 reads [a]		55	31
No of genes showing no differential expression [b]		33	2
No of genes over-expressed during the infectious cycle and their cluster assignations [c] :		22	29
	cluster 1	0	1
	cluster 2	6	26 (including 7 *AvrLm* genes)
	cluster 3	0	1
	cluster 4	2	0
	cluster 5	0	0
	cluster 6	0	1
	cluster 7	5	0
	cluster 8	9	0

B

Gene categories		Non-SSP	SSP
Total number of genes with a count > 10 reads [a]		331	62
No of genes showing no differential expression [b]		278	11
No of genes over-expressed during the infectious cycle and their cluster assignations [c] :		53	51
	cluster 1	6	2
	cluster 2	27	40 (including 6 *AvrLm* genes)
	cluster 3	2	2
	cluster 4	8	3
	cluster 5	9	4 (including one late effector)
	cluster 6	0	0
	cluster 7	1	0
	cluster 8	0	0
