# Supplementary material for: Large-scale transcriptomics to dissect 2 years of the life of a fungal phytopathogen interacting with its host plant
Source: BMC Biol. 2021 Mar 23;19:55. doi: 10.1186/s12915-021-00989-3 (PMC7986464; doi:10.1186/s12915-021-00989-3)
Supplement: Supplementary file 23 — Additional file 23: Table S8. Conservation of the protein sequences encoded by highly co-expressed SSP genes. [file 12915_2021_989_MOESM23_ESM.pdf]

S8 Table. Conservation of the protein sequences encoded by highly co-expressed SSP genes.

[a.] Number of SSP genes detected as highly correlated with each reference expression wave

[b.] Number of *AvrLm* effectors and *LmSTEE* effectors detected as highly correlated with each reference expression wave

[c.] Number of SSP sequences with no homology after a BLAST search

Assignation to wave	[a.] Total no of highly co-regulated SSP genes	[b.] No of known effectors in *L. maculans*	[c.] No of SSP without homologous protein in the non-redondant protein NCBI database
Wave 1	1	-	-
Wave 2	39	7	23
Wave 3	0	-	-
Wave 4	20	3	12
Wave 5	7	2	2
Wave 6	5	0	1
Wave 7	23	0	2
Wave 8	1	-	-
